# On-Demand Cross-Linkable Bottlebrush Polymers for Voltage-Driven Artificial Muscles

**DOI:** 10.1021/acsami.2c23026

**Published:** 2023-04-12

**Authors:** Yeerlan Adeli, Francis Owusu, Frank A. Nüesch, Dorina M. Opris

**Affiliations:** † Laboratory for Functional Polymers, Swiss Federal Laboratories for Materials Science and Technology Empa, Ueberlandstr. 129, CH-8600 Dübendorf, Switzerland; ‡ Institute of Chemical Sciences and Engineering, Ecole Polytechnique Federale de Lausanne, EPFL, Station 6, CH-1015 Lausanne, Switzerland

**Keywords:** artificial muscles, actuators, dielectrics, bottlebrush polymers, electrically responsive elastomers, ROMP

## Abstract

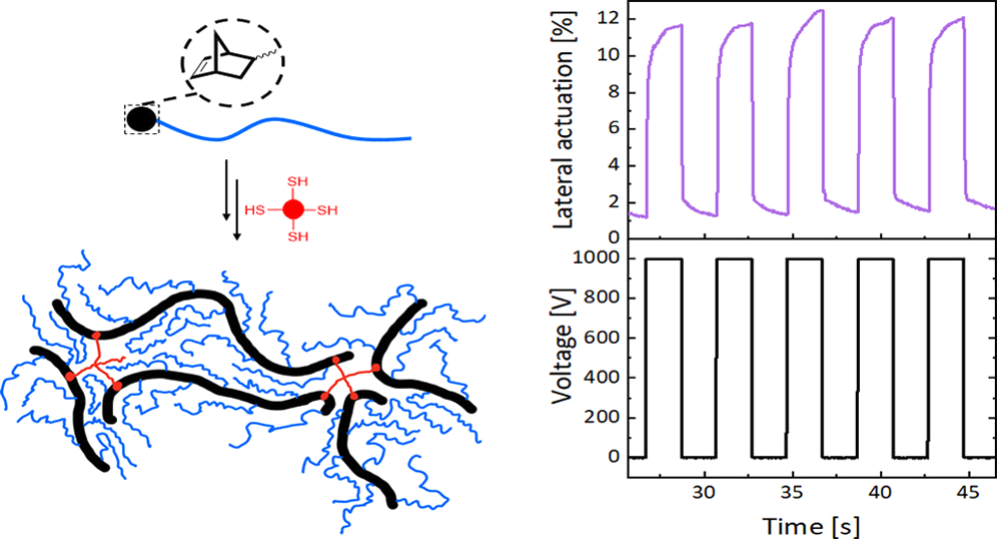

Dielectric elastomer actuators (DEAs) generate motion resembling natural muscles in reliability, adaptability, elongation, and frequency of operation. They are highly attractive in implantable soft robots or artificial organs. However, many applications of such devices are hindered by the high driving voltage required for operation, which exceeds the safety threshold for the human body. Although the driving voltage can be reduced by decreasing the thickness and the elastic modulus, soft materials are prone to electromechanical instability (EMI), which causes dielectric breakdown. The elastomers made by cross-linking bottlebrush polymers are promising for achieving DEAs that suppress EMI. In previous work, they were chemically cross-linked using an in situ free-radical UV-induced polymerization, which is oxygen-sensitive and does not allow the formation of thin films. Therefore, the respective actuators were operated at voltages above 4000 V. Herein, macromonomers that can be polymerized by ring-opening metathesis polymerization and subsequently cross-linked via a UV-induced thiol–ene click reaction are developed. They allow us to fast cross-link defect-free thin films with a thickness below 100 μm. The dielectric films give up to 12% lateral actuation at 1000 V and survive more than 10,000 cycles at frequencies up to 10 Hz. The easy and efficient preparation approach of the defect-free thin films under air provides easy accessibility to bottlebrush polymeric materials for future research. Additionally, the desired properties, actuation under low voltage, and long lifetime revealed the potential of the developed materials in soft robotic implantable devices. Furthermore, the C–C double bonds in the polymer backbone allow for chemical modification with polar groups and increase the materials’ dielectric permittivity to a value of 5.5, which is the highest value of dielectric permittivity for a cross-linked bottlebrush polymer

## 1 Introduction

Artificial muscles are soft actuators capable of generating force and shape change when exposed to external stimuli, such as pH, temperature, humidity, and electric and magnetic fields.^1^ However, the force and the strain generated by most soft actuators are too small to be useful as artificial muscles. Tremendous research has been invested in optimizing the generated stress and strain, energy density, and lifetime to closely mimic natural muscle.^2−8^ Such a match will allow one-day malfunctioning muscle replacement by an artificial one. Additionally, the artificial muscles should be easily operable and produce no adverse effects on the body during operation. Elastomers have many characteristics that make them resemble natural muscles. They reversibly change shape when mechanically stressed and their elastic modulus can be easily tuned from a few kPa to MPa ranges to match the properties of any natural muscle.^9^ This material property will impact the external force needed to induce deformation. One technology closely emulating natural muscles is dielectric elastomer actuators (DEA). They are soft capacitors that deform when electrically charged and recover the initial shape when discharged due to the elastic restoring forces.^10^ The controllable strain, force, response frequency, and the silent deformation^2^ made the DEAs emerge in many novel applications,^11,12^ including implantable soft robots.^13^ Those devices can potentially support malfunctioning muscles or replace failed organs such as artificial hearts. The DEAs could be used for such purposes, but they should be reliable, compact, durable, and adaptable to generate the desired motion. Additionally, they should be operable at low voltages. However, existing DEAs do not meet the safety standard for such applications due to the high driving voltage.^14^

Pelrine’s equation (eq 1), where P is the electrostatic pressure, ε’ is the dielectric permittivity, *U* is the voltage between the two electrodes, *d* is the distance between two electrodes and ε_0_ is the constant, valid for thickness strains (*s*_z_) below 10%, suggests three ways achieve this: increase ε’, reduce the elastic modulus (*Y*), and decrease the dielectric film thickness (*d*)^3^
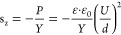
1However, soft materials are prone to electromechanical instability (EMI).^15^ Under an external electric field, a soft dielectric film thins down, leading to an enhanced electric field, which contributes to the further thinning of the film and ends up with a breakdown. The ideal material for DEAs is soft at small strains allowing for achieving the desirable actuation and stiff at larger strains, preventing further film thinning, thus overcoming EMI.

Several approaches have been used to achieve materials with favorable stress–strain behavior. They include swelling the network with a solvent, pre-straining the network, and synthesizing interpenetrating networks.^16^ The first has the disadvantage of solvent leaking and aging,^17^ while the second needs a rigid frame to keep the strain, which increases the device’s size. The third has the advantage that the rigid frame can be eliminated^18^ by forming a second network, which keeps the pre-strain in the device. However, this needs an additional step, which slows the manufacturing process and increases the costs.

Recently, elastomers made by cross-linking bottlebrush polymers seem promising.^19−23^ Bottlebrush polymers have a polymer backbone whose repeat units carry polymer side chains.^24^ The side chains keep the backbone stretched, rendering fewer entanglements.^25^ The low amount of chain entanglements and the plasticizing effect of the side chains make the bottlebrush polymer materials soft at small strains. In contrast, strain stiffening increases the elastic modulus at large strains.^26^ Their unusual stress–strain behavior allows bottlebrush polymer materials to respond to low voltages and prevents them from entering EMI at higher voltages.

Several examples of dielectric elastomers prepared by physical^19^ or chemical cross-linking of bottlebrush polymers have been reported.^20−23^ Block copolymers consisting of soft bottlebrushes and hard polystyrene segments allow the formation of physically cross-linked soft elastic materials. Chemically cross-linked bottlebrush polymers were synthesized by in situ polymerization in thin films of a macromonomer and a multifunctional monomer that functions as a cross-linker.^19^ Thus, polymer side chains are simultaneously grafted to a polymer backbone and cross-linked^22^ typically by a radical initiator.^20^ Though the performance of the physically cross-linked bottlebrush polymers presents superior combinations of softness and strain stiffening than their chemically cross-linked counterparts,^19^ the former may be less favorable in low-frequency applications and high strains because the reversible cross-links can break at lower strain rates.^27,28^ Accordingly, under certain working frequencies and strains, the physical cross-links may weaken and vanish through the dissipation of strain energy, negatively impacting elasticity. On the contrary, chemically cross-linked bottlebrush materials show more stable properties in a large frequency range and are more suitable for applications requiring various frequencies and strains. An actuator constructed from a chemically cross-linked bottlebrush polymeric material gave an areal actuation above 300% under an electric field of 10 V/μm. However, because the dielectric films used were 440 μm thick, the driving voltage was above 4000 V.^19^

Although it may look easy to bridge the gap between the low electric field and actuation at a low voltage by simply reducing the film thickness, this step is challenging. For instance, the reported bottlebrush polymers were prepared by a free-radical-based UV-induced polymerization, which required a mold to prevent oxygen inhibition.^29^ The transparent mold allowed the formation of 1 mm thick films, which are much too thick for actuator application. A reduction of film thickness by a mold can theoretically be achieved; however, the adhesion forces between the films and the mold are so strong that it is practically impossible to get the soft film out of the mold without rupturing it. Additionally, continuous manufacturing of devices cannot be achieved.^29^ Furthermore, the in situ polymerization/cross-linking was achieved by UV light irradiation for 12 h, which is neither economical nor environmentally friendly and is not suitable for a continuous manufacturing process of actuators. Hence, finding an efficient, simple, robust, and green way to prepare high-quality, defect-free bottlebrush-based thin films is crucial.

Herein, we overcome these problems using specially designed monomers that can be efficiently polymerized and cross-linked under a normal environment. The bottlebrush polymers were synthesized by ring-opening metathesis polymerization starting from a norbornene macromonomer modified with a poly(dimethylsiloxane) chain. The synthesized bottlebrush polymers have double bonds in their backbone, which are subsequently used for cross-linking into thin films by the fast, efficient, reliable, and air-insensitive thiol–ene reaction.^30−32^ This allowed us to produce defect-free thin films of chemically cross-linked bottlebrush polymers in the air. Besides thoroughly investigating materials’ thermal stability and dielectric and mechanical properties, the electromechanical response was also investigated. The dielectric material was capable of up to 12% lateral actuation at 1000 V. Additionally, we showed that the chemically cross-linked bottlebrush polymeric materials could be actuated many times at frequencies up to 10 Hz and survived more than 10,000 cycles. Such tests are rarely reported but are necessary when considering applications.

## 2 Results and Discussion

**Scheme 1:**
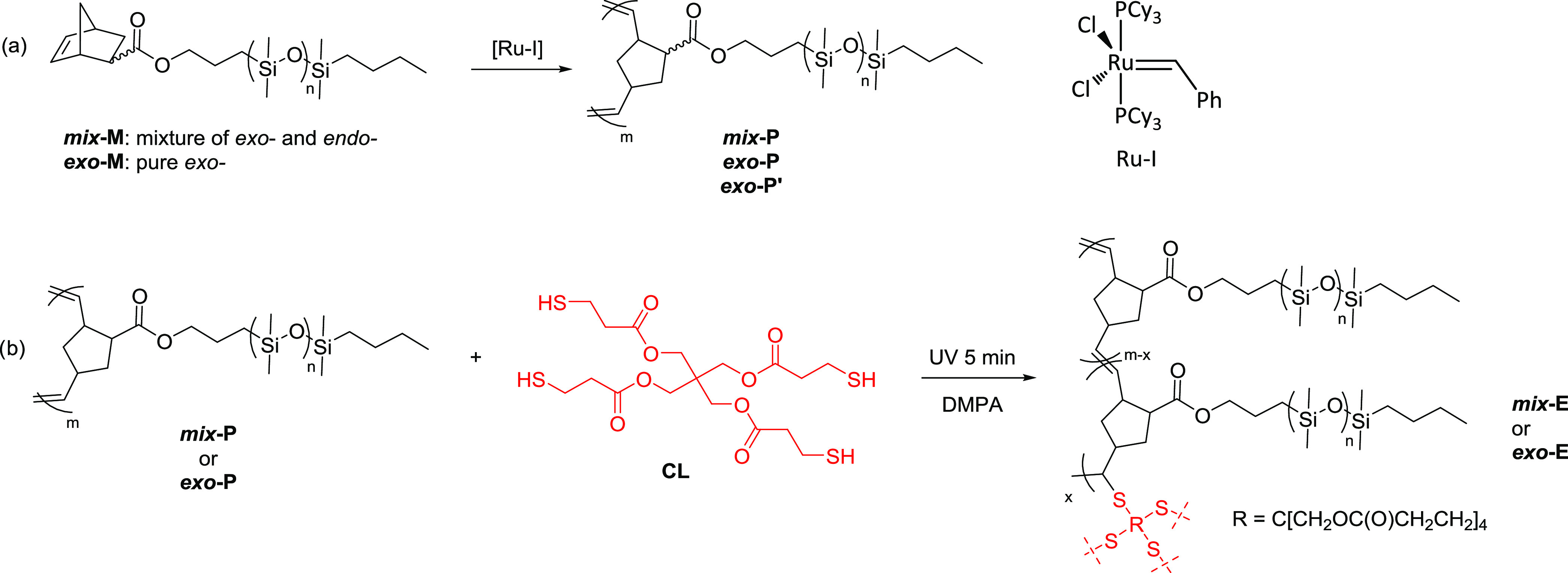
Synthetic Path to Bottlebrush Elastomers via Ring-Opening Metathesis Polymerization of Macromonomer ***mix*****-M** or ***exo*****-M** (a) and Subsequent Cross-Linking by the Thiol–Ene Reaction of Pentaerythritol Tetrakis(3-Mercaptopropionate) (CL) with Double Bonds from ***mix*****-P** and ***exo*****-P** Backbone to form Elastomers ***mix*****-E** and ***exo*****-E**, Respectively (b)

The synthetic strategy to chemically cross-linked bottlebrush polymers is illustrated in Scheme 1. A mono-hydroxyl-terminated poly(dimethylsiloxane) (PDMS) (*M*_n_ = 1136 g/mol as determined by ^1^H NMR end group analysis) was prepared by a hydrosilylation reaction between allyl alcohol and a commercial monohydride-terminated PDMS (Figure S1). The norbornene poly(dimethylsiloxane) macromonomers were synthesized by an esterification reaction of a mixture of *exo* and *endo* (mix) isomers or pure *exo*-norbornene carboxylic acid and the mono-hydroxyl-terminated poly(dimethylsiloxane) (PDMS) to give the macromonomer ***mix*****-M** (*M*_n_ = 1226 g/mol, by ^1^H NMR end group analysis) and ***exo*****-M** (*M*_n_ = 1508 g/mol, by ^1^H NMR end group analysis). The ^1^H and ^13^C NMR spectra of the monomers show that the esterification was successful (Figures S2 and S3).

**Figure 1:**
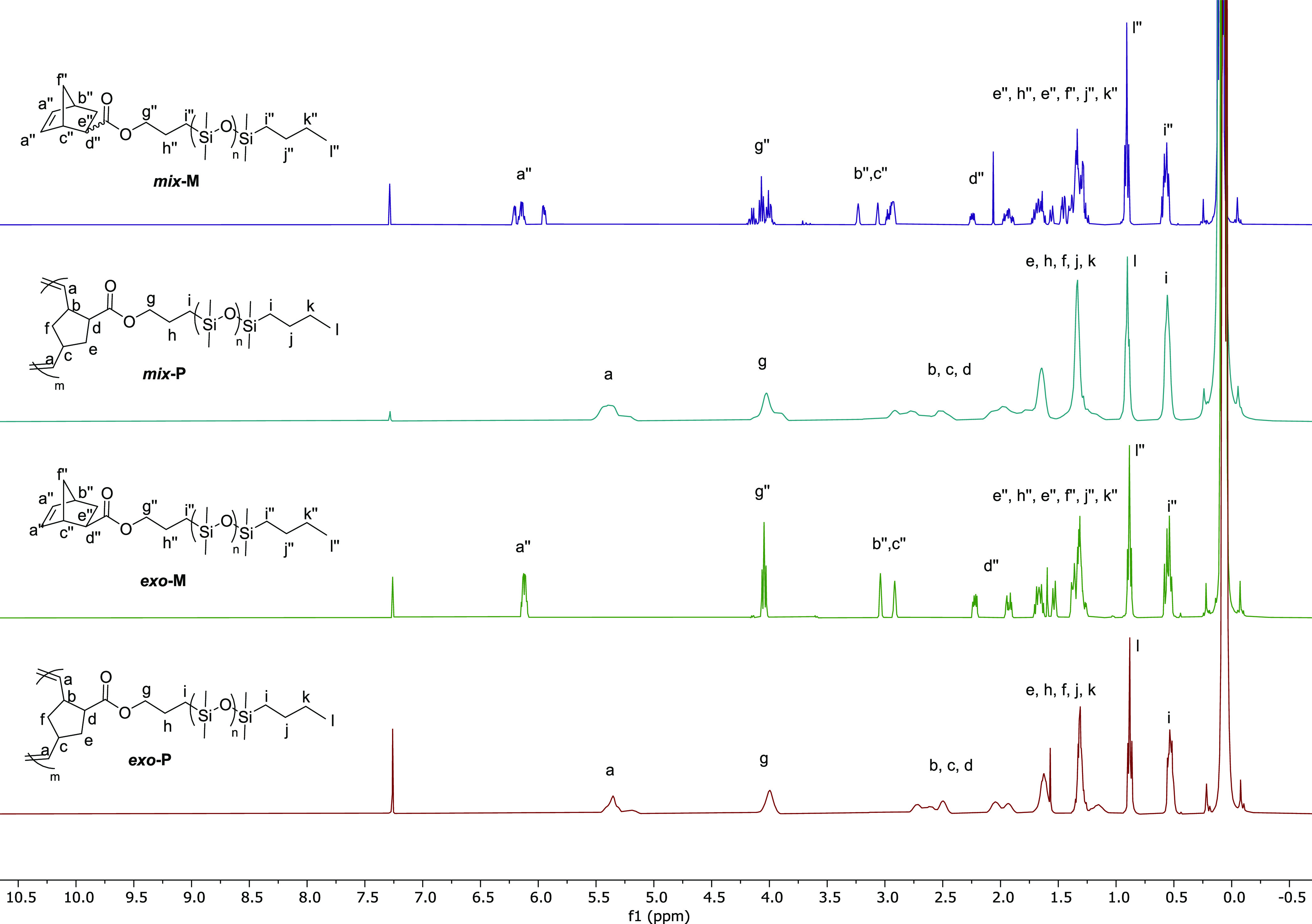
^1^H NMR spectra of macromonomer ***mix*****-M** and polymer ***mix*****-P** (top) and macromonomer ***exo*****-M** and polymer ***exo*****-P** (bottom).

Ring-opening metathesis polymerization (ROMP) of ***exo*****-M** and ***mix*****-M** using first-generation Grubb’s catalysis afforded bottlebrush polymers ***exo*****-P**, ***exo*****-P′**, and ***mix*****-P** (Scheme 1**a**). The molar ratio of monomer to initiator was kept constant at 800:1. The success of the polymerization was proven by ^1^H NMR spectroscopy, which shows a proton chemical shift displacement of the vinylene group between 6.5 and 6.0 ppm to 5.6 and 5.1 ppm for the monomers and polymers, respectively (Figures 1 and S4–S6).

The molar mass and polydispersity index (PDI) of the polymers were determined by gel permeation chromatography (GPC) using PDMS standards (Table S1 and Figure S7). Polymer ***mix*****-P** showed an *M*_n_ = 23.6 kDa and a PDI = 1.61, while ***exo*****-P** and ***exo*****-P′** showed an *M*_n_ = 33.2 kDa and a PDI 2.24 and an *M*_n_ = 271.2 kDa and a PDI = 2.76, respectively (Table 1). Because of the more compact structure of polynorbornene bottlebrush polymers compared to PDMS standards, the molecular weight of the former is underestimated and thus should be taken with great care.^33,34^

**Table 1:** Molar Mass and Molar Mass Distribution of the Polymers

entry	monomer configuration	polymerization time^a^	*M*_n_ [kDa]	*M*_w_ [kDa]	PDI
***mix*****-P**	*exo*/*endo*	12 h^b^	23.6	38.1	1.61
***exo*****-P**	*exo*	2 h^c^	33.2	74.4	2.24
***exo*****-P′**	*exo*	3.5 h^c^	271.2	750.0	2.76

a The molar ratio of monomer: catalyst = 800:1.

b Reflux at 65 °C.

c Heating at 40 °C.

High-molar-mass bottlebrush polymers are needed to achieve materials with good elastic properties.^35^ The ***exo*****-M** polymerized faster and gave higher-molar-mass polymers than its counterpart since the ROMP favors *exo*- over the *endo*-macromonomer (2 h for ***exo*****-P** vs 12 h for ***mix*****-P**) (Table 1).^36^ Although macromonomer ***mix*****-M** was less reactive and needed longer polymerization time, it was nevertheless further used because the starting *exo-*/*endo-*5-norbornene-2-carboxylic acid is cheaper than the *exo*-5-norbornene carboxylic acid. The rather large PDI of the ***exo*****-P** and ***exo*****-P**′, indicate that side reactions occurred, likely toward the end of the polymerization, which led to a higher dispersity. At the same time, the less reactive ***mix*****-M** monomers led to a lower polymerization rate, which may be responsible for the narrower PDI of ***mix*****-P**.

ROMP affords polymers with C–C double bonds in their backbone, which can be used for cross-linking.^36^ Thin films were prepared by doctor blading and cross-linked by UV-induced thiol–ene reaction with pentaerythritol tetrakis(3-mercaptopropionate) cross-linker (CL) in the presence of 2,2-dimethoxy-2-phenylacetophenone (DMPA) photoinitiator. Because the thiol–ene reaction is fast, the cross-linking is completed within 5 min. Compared to other free-radical-induced cross-linking protocols, oxygen interference is less significant for the thiol–ene reaction, which can be performed in the air.^21,37,38^ This makes the entire process easier, cheaper, and scalable. It also does not require molds that can make it utterly difficult to accomplish thin films with no defects. Additionally, the ratio between the double bonds and the thiol from CL can be easily tuned, which allowed us to synthesize a series of thin films with tunable mechanical properties (Table 2). The materials were named ***mix*****-E**_**n**_ or ***exo*****-E**_**n**_, where n represents the molar concentration of the thiol group of the CL to the mass of the polymer backbone. Toluene was used to facilitate the mixing of different components. Thin films were made by doctor blading and the solvent was left to evaporate for 8 hours. Thereafter the films were cross-linked by exposing them to UV light for 5 min. Only films made of materials ***exo*****-E**_**62**_**′** and ***exo*****-E**_**50**_**′** were cross-linked immediately after casting. Infrared spectroscopy (IR) characterization showed the signals from the key functional groups; however, quantitative correlations between the peaks were not obvious (Figure S8). In solution, the bottlebrush polymer is more stretched and the number of entanglements is reduced.^35,39^ Although bottlebrush polymers have a lower tendency to give chain entanglements,^33^ the effect cannot be overlooked for materials prepared from ***exo*****-P** polymers that have higher molar masses.

**Table 2:** Mechanical Properties of ***mix*****-E**_**n**_ and ***exo*****-E**_**n**_ Silicone Elastomers Sample^c^

entry	polymer used	–SH^a^ [μmol/g]	–SH:double bonds^c^	*Y*_10%_ [kPa]^d^	*Y*_50%_ [kPa]^e^	*Y*_100%_ [kPa]^f^	s [%]^g^	gel fraction [%]
***mix*****-E**_**62**_	***mix*****-P**	62	0.058	82 ± 11	66 ± 5		84 ± 8	88
***mix*****-E**_**41**_	***mix*****-P**	41	0.039	55 ± 8	47 ± 5	46 ± 4	120 ± 48	70
***mix*****-E**_**20**_	***mix*****-P**	21	0.019	29 ± 6	24 ± 3	22 ± 3	149 ± 37	47
***exo*****-E**_**62**_	***exo*****-P**	62	0.058	30 ± 3	32 ± 1	31 ± 1	134 ± 22	82
***exo*****-E**_**62**_**′**^b^	***exo*****-P**	62	0.058	19 ± 5	20 ± 6	21 ± 6	125 ± 23	85
***exo*****-E**_**50**_	***exo*****-P**	50	0.047	27 ± 11	27 ± 8		90 ± 8	82
***exo*****-E**_**50**_**′**^b^	***exo*****-P**	50	0.047	11 ± 1	14 ± 1	14 ± 1	291 ± 80	80

a Concentration of the thiol functional group to the mass of the polymer used.

b Polymer cross-linked with solvent.

c Molar ratio.

d Elastic modulus at 10% strain.

e Elastic modulus at 50% strain.

f Elastic modulus at 100% strain.

g Average strain at break of five samples.

**Figure 2:**
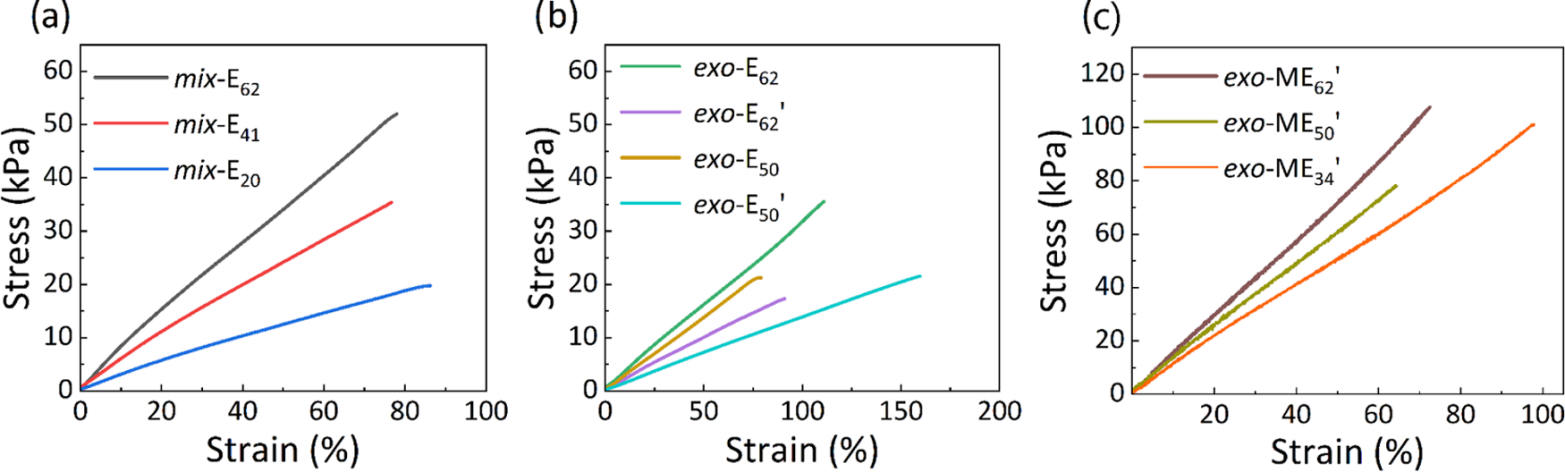
Stress–strain curves for materials ***mix*****-E**_**n**_ (a), ***exo*****-E**_**n**_ (b), and ***exo*****-ME**_**n**_**′** (c). Tensile tests were conducted on five replicates per material, and for each material, the average was given. The data obtained were analyzed using Origin. Therefore, the strain at break in each curve represents the lowest strain at break from the replicates tested per material. For the original tensile curves of each replicate per material, see Figure S9.

Tensile tests were conducted on dumbbell-shaped samples (Figures 2 and S9 and S10). Figure 2 gives the average stress–strain curves of at least five different samples measured for each material, while the elastic modulus at different strain levels was calculated from the slope of the curves at different strain levels (Table 2). The elastic modulus of all materials was below 100 kPa, which confirms the softening effect of the brushes. Most materials have a high gel fraction of above 80%, except for ***mix*****-E**_**20**_ where the extractible was over 50%, which may indicate a poorer cross-linking, although soft materials can easily break during swelling due to the large tension along network strands.

An ideal elastomer for DEA will be soft at small strains and stiffen at large strains preventing the actuator from EMI. Unfortunately, the strain stiffening of our materials was not observed for ***mix*****-E**_**n**_ and was limited for ***exo*****-E**_**n**_.^29,40^ According to Haugan, the norbornene backbone polymer chains are prone to strong interaction, giving helical structures.^41,42^

Material ***mix*****-E**_**62**_ was excluded from further investigations because its elastic modulus dropped from 82 kPa at 10% strain to 66 kPa at 50% strain. Also for ***mix*****-E**_**41**_, the elastic modulus decreased from 55 to 47 kPa at 50% strain, but the softening effect was slightly less pronounced than for ***mix*****-E**_**62**_. The materials **exo-E**_**62**_′ and **exo-E**_**50**_**′** showed an almost negligible increase in the elastic modulus with strain. Thick films (over 400 μm) made from ***mix*****-E**_**21**_ showed more favorable mechanical properties than ***mix*****-E**_**41**_; however, films with a thickness below 100 μm did not cross-link. Therefore, this sample was not considered for further tests.

This is likely due to the oxygen scavenging, which may be more pronounced for this sample as it uses the smallest amount of thiol. Although thiol–ene reaction is truly efficient and allows cross-linking in air, its mechanism is free-radical-based. The oxygen in the air can react with free radicals and form oxygen-centered radicals, which can abstract hydrogen from thiol by forming thiyl radical. Hence, the concentration of thiol cross-linker is critical since it not only participates in the cross-linking reaction but also donates hydrogen. This effect is more pronounced in thin films and low concentrations of thiol.^29^

Comparing the mechanical properties of ***exo*****-E**_**62**_′ and ***exo*****-E**_**50**_**′** cross-linked in the solution immediately after casting in thin films with those of ***exo*****-E**_**62**_ and ***exo*****-E**_**50**_, cross-linked after the solvent was allowed to evaporate, revealed that ***exo*****-E**_**62**_**′** and ***exo*****-E**_**50**_**′** are softer compared to ***exo*****-E**_**62**_ and ***exo*****-E**_**50**_, though the same amounts of reagents were used, suggesting that cross-linking in solvent reduces entanglements.

Thermogravimetric analysis (TGA) was used to assess the materials’ thermal stability from 0 to 600 °C under an inert atmosphere (Figure S11). The materials were stable up to 300 °C, and the first loss peak appeared above 350 °C.

**Figure 3:**
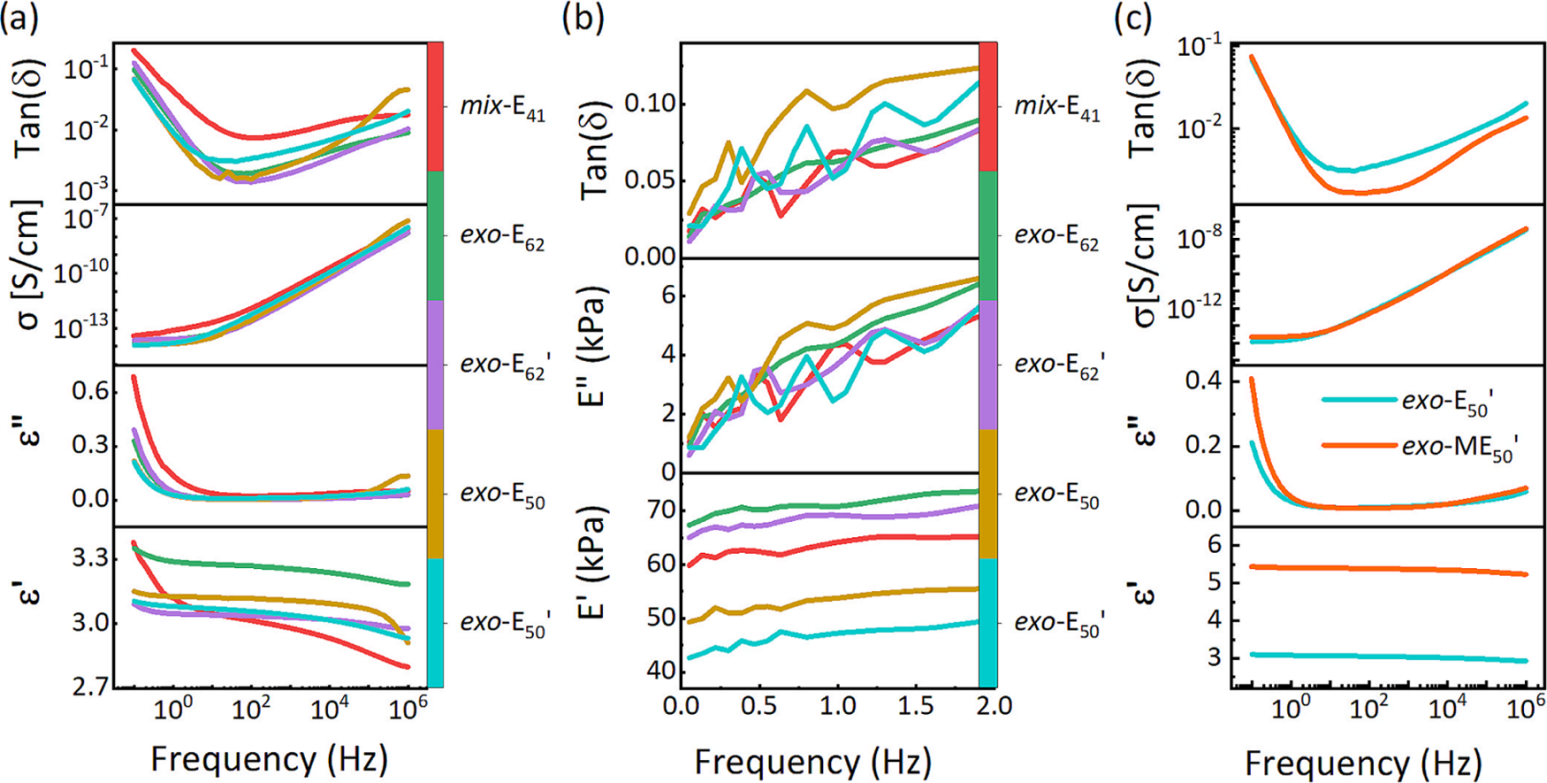
Dielectric permittivity (ε′), dielectric loss (ε″), loss factor (tan δ), and conductivity (σ) of the materials as a function of frequency at room temperature (a), dynamic mechanical response of different materials at frequencies ranging from 0.05 to 2 Hz and 2% strain (b), and dielectric permittivity (ε′), dielectric loss (ε″), loss factor (tan δ), and conductivity (σ) of the ***exo*****-E**_**50**_**′** and ***exo*****-ME**_**50**_**′** materials as a function of frequency at room temperature (c).

We further investigated the dielectric properties by impedance spectroscopy (Figure 3a). Due to the limited content of polar ester groups, all materials showed a moderate permittivity ranging from 2.6 to 3.3. The dielectric response of ***mix*****-E**_**41**_ was slightly different from the other four samples where *exo* monomer was used. It is known that polymers with certain regioisomers, stereochemical configuration, and tacticity give a different dielectric response.^43^ Materials ***exo*****-E**_**62**_**′** and ***exo*****-E**_**50**_**′** had a slightly lower permittivity than materials ***exo*****-E**_**62**_ and ***exo*****-E**_**50**_, which may be due to more free volume created after solvent removal from the cross-linked films of the former and a more compact packing for the later. Besides, the tan δ (ε″/ε’ ratio) indicates that the dielectric materials’ best-operating frequency is between 10 and 100 Hz. At these frequencies, the *tan*δ values are below 0.01, and thus heating caused by dipole relaxation or ion motion is minimized. This suggests the most efficient working frequency window, which needs to be considered. The heat generated during actuation is especially important in stack actuators, where the heat cannot be easily transferred to the environment and the device may fail due to overheating. Besides this, the conductivity at low frequency is below 5 × 10^–14^ S/cm, which qualifies these materials for dielectric applications.

Moreover, the samples were investigated by dynamic mechanical analysis (DMA) (Figure 3b). At least three stripe-shaped samples per material with a size of 1.5 cm × 2 cm were independently analyzed at room temperature at a strain of 2%. The values, including storage modulus *E*′, loss modulus *E*″, and mechanical loss factor tan δ were averaged and plotted. The tested materials showed almost constant storage modulus over the investigated frequencies ranges between 50 mHz and 2 Hz. All materials were rather soft and showed a storage modulus below 75 kPa. Besides, the loss factors remained small, below 0.15, confirming the materials’ good elastic properties. Low mechanical losses are important for achieving actuators with small hysteresis in cyclic actuation tests.

**Figure 4:**
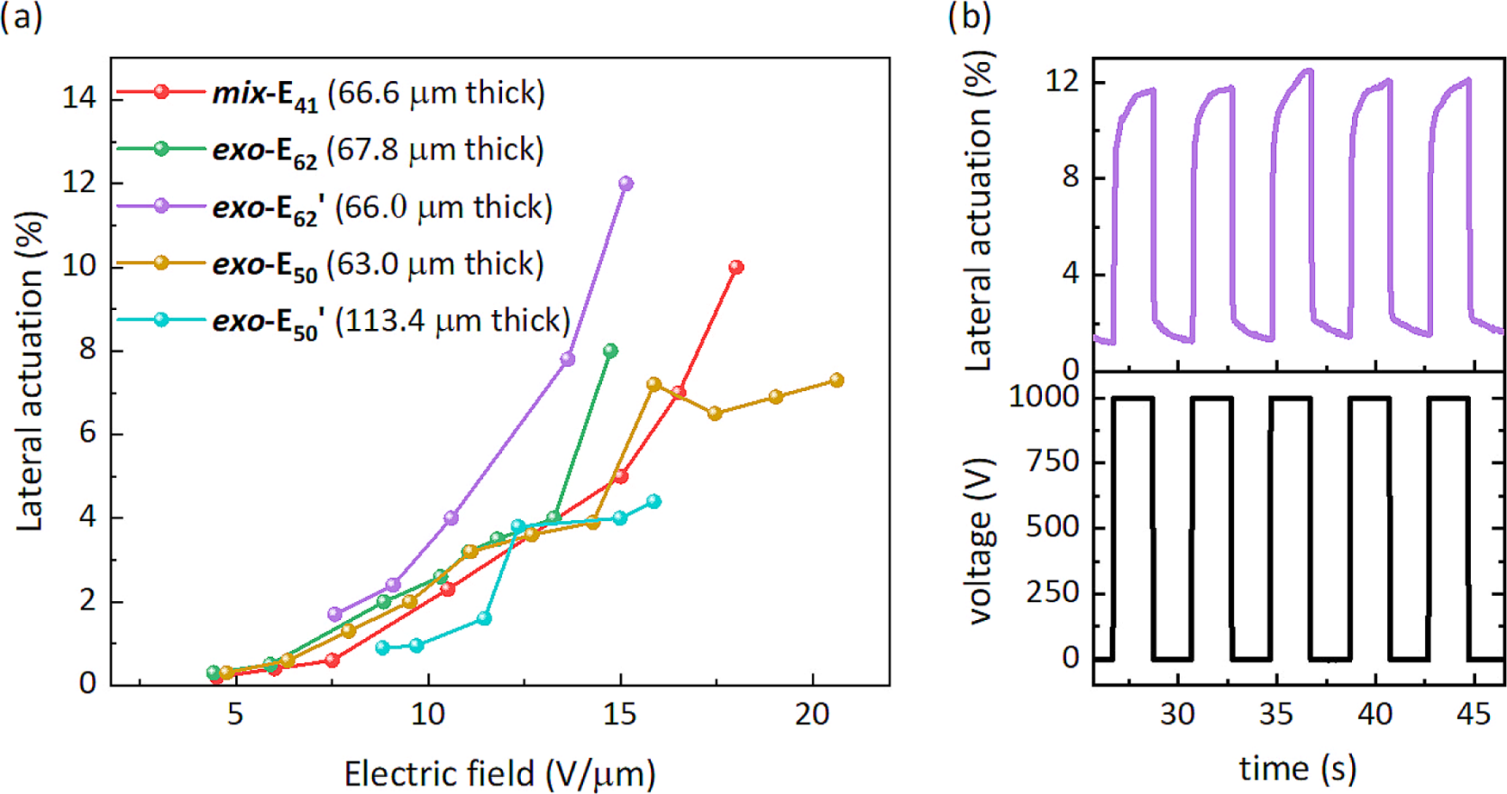
Lateral actuation strains of free-standing thin films under different electric fields (a) and 5 out of 10 cyclic actuation steps at 0.25 Hz of material ***exo*****-E**_**62**_**′** 66 μm thick (b).

After confirming the materials’ potential through mechanical and dielectric investigations, we turned our attention to the electromechanical responses. Circular actuators were constructed by fixing the defect-free thin films with no pre-strain between a pair of rigid frames with an inner and outer diameter of 25 and 30 mm, respectively. Carbon black powder was applied on both sides of the dielectric film to form a circular capacitor with a diameter of 8 mm. Figure 4 summarizes the performances of the actuators under different electric fields. Because the materials were rather soft, they actuated at a low electric field below 20 V/μm. The best performance in the actuator test was observed for ***exo*****-E**_**62**_**′**. It gave a lateral actuation of 12% (25% area actuation strain) at 15.2 V/μm (Figure 4a). This actuator was subjected to 10 actuation cycles at 0.25 Hz, and after five cycles, the actuation stabilized to 12% and was reversible (Figure 4b). The stiffest material, ***mix*****-E**_**41**_ gave 10% lateral actuation (21% actuation area) at 18 V/μm. Although those achievements look inferior to other bottlebrush polymers reported,^20^ our material can be actuated at 1000 V. In contrast, the reported one gave the same actuation at 2112 V for a much softer material (*Y*_10%_ = 1.5 ± 0.2 kPa). Additionally, all reported films made by chemically cross-linking bottlebrush polymers were so thick that the actuators constructed responded to a much higher voltage than those of this work.

Besides the efficient and easy accessibility of defect-free thin films, the best bottlebrush dielectric material presented in this work gave a 12% lateral actuation at 1000 V (about 15 V/μm) (Figure 4). Thus, we achieved defect-free thin free-standing film with an ultralow elastic modulus, achieving responsive actuators at much lower voltages. At the same time, owing to the stiffening effect induced by the bottlebrushes, most materials’ breakdown field strength (*E*_b_) was above 20 V/μm (Table 3), which is about 2 times higher than regular elastomers with similarly low elastic moduli.^44^ The measured values of *E*_b_ are of the same order of magnitude as the ones predicted for homogeneous elastomers calculated using the formula derived by Stark and Garton^45−47^
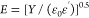
2which were included in Table 3. This shows that polymer films behave as perfect elastomers and are not prone to structural inhomogeneities. Furthermore, the films can be activated up to their maximum lateral strain without suffering from electromechanical instability.

**Table 3:** Maximum Lateral Actuation Strain (*s*_max_), Dielectric Breakdown of the Actuator (*E*_b,act_), and the Original Film Thickness (*d*_0_)

entry	*s*_max_^a^ [%]	*s*_max-areal_^b^ [%]	voltage @ *s*_max_^c^ [V]	*d*_0_^d^ [μm]	Δ*d*^e^ [μm]	*E*_b,act_^f^ [V/μm]	ε′	*Y*_10%_ [kPa]	*E*_b_,_calculated_^g^ [V/μm]
***mix*-E**_**41**_	10.0	21.0	1200	66.6	11.6	21.8	2.94	55 ± 8	46.0
***exo*-E**_**62**_	7.0	14.5	1200	67.8	8.6	20.3	3.24	30 ± 3	32.3
***exo*-E**_**62**_**′**	12.1	25.7	1000	66.0	13.5	20.9	3.02	19 ± 5	26.7
***exo-*E**_**50**_	7.0	14.5	1300	63.0	8.2	23.7	3.09	27 ± 11	31.4
***exo*-E**_**50**_**′**	4.4	9.0	1800	113.4	9.4	17.3	3.02	11 ± 1	20.3

a Maximum lateral strain.

b Maximum areal strain.

c Voltage at the highest lateral actuation.

d Original thickness of the thin film.

e Change in thickness Δ*d* = *d*_0_ – *d*, considering the corresponding thickness *d*: *d* = *d*_0_/(*s*_max_ + 1)^2^.

f Exact breakdown fields of the thin film given by *E*_b,act_ = [voltage @ *s*_max_]/*d*.

g Calculated breakdown fields of the thin film according to the theory by Stark and Garton, taking *Y*_10%_ and ε′, permittivity of the materials at 10 kHz.^45,46^

**Figure 5:**
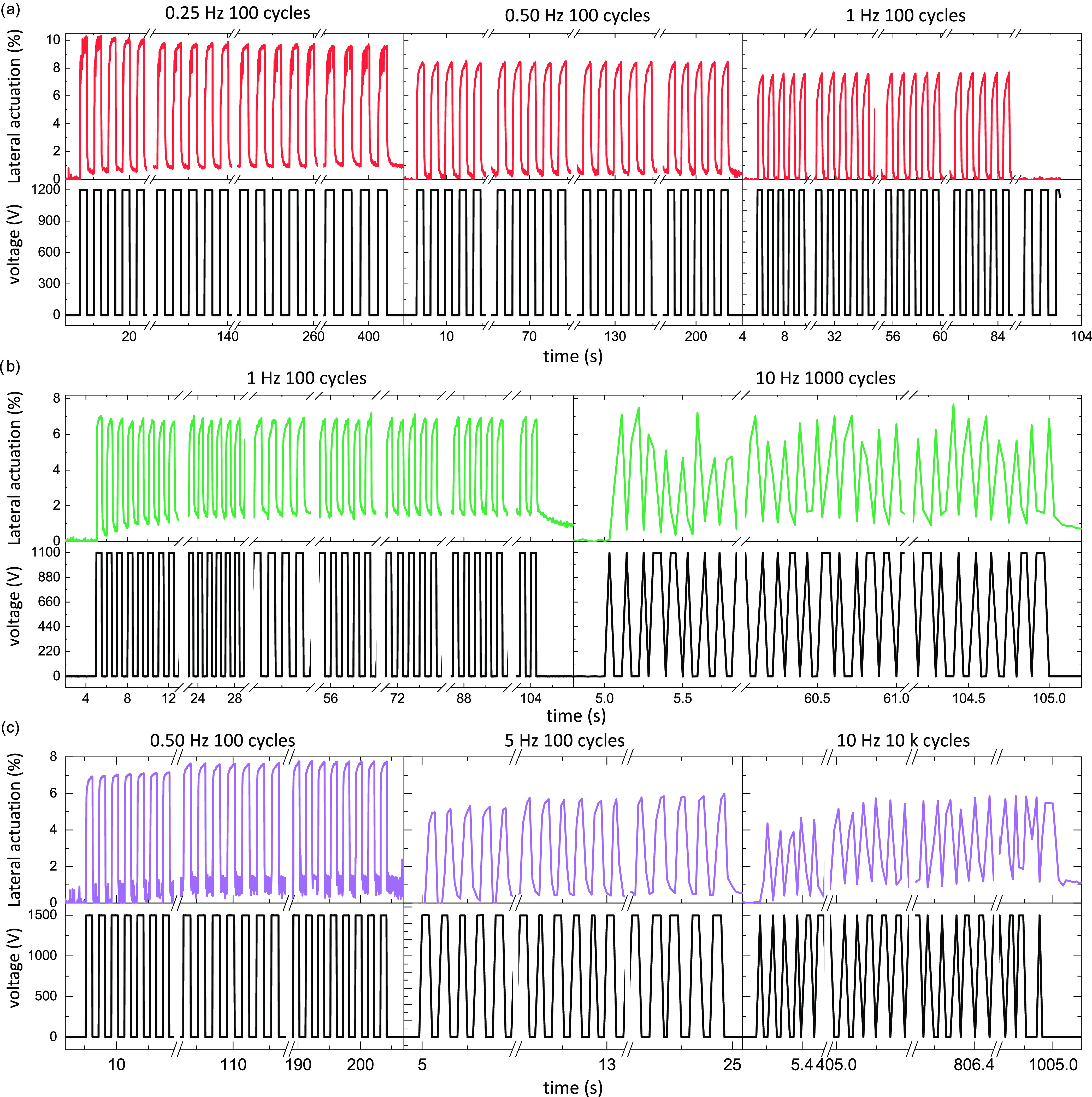
Cyclic DEA measurements of the free-standing thin films over 100 cycles: (a) material ***mix*****-E**_**41**_ (film thickness of 66.6 μm, electric field of 18 V/μm); (b) ***exo*****-E**_**62**_ (film thickness of 74.3 μm, electric field of 15 V/μm); and (c) ***exo*****-E**_**62**_**′** (film thickness of 89.0 μm, electric field of 17 V/μm). The noisy values at 10 Hz are caused by the fact that the software cannot record sufficient data due to the fast actuation. The ununiform response of actuators at 10 Hz is not due to material but rather a software problem, as insufficient points are measured.

Apart from electromechanical tests, the reliability of our materials was evaluated in cyclic tests at different frequencies (Figure 5) with unprestrained actuators. For real applications, the lifetime of the dielectrics is probably even more important than other characterization parameters. For instance, implanted soft robotic devices ought to be durable and operable for a considerable time to minimize the surgery relating to maintenance or replacements. Only a few works report on the lifetime of actuation tests over 100 cycles.^48^ Here, the actuation lifetime at different frequencies was investigated. Since the measurement was conducted using different actuators with varying film thicknesses, the actuation voltages vary from those shown in the previous figures or tables. The pattern at a defined working frequency can be illustrated by taking the frequency of 0.5 Hz as an example. First, we set the voltage on for one second and off for another second. As demonstrated (Figure 5a, middle), because of its low mechanical loss factor tan δ, material ***mix*****-E**_**41**_ gave a reversible actuation at 18 V/μm for almost 300 cycles. At a frequency of 0.25 Hz, the material gave 10% lateral actuation strain without obvious decay for at least 100 cycles. With increasing the frequency, a slight decrease in actuation was observed from 8.5% at 0.5 Hz to 7.5% at 1 Hz. At the investigated frequencies, no hysteresis in actuation was observed, which supports the excellent elasticity of these materials.

As mentioned before, materials ***exo*****-E**_**62**_ (*Y*_10%_ = 30 ± 3 kPa) and ***exo*****-E**_**62**_**′** (*Y*_10%_ = 19 ± 5 kPa) were made starting from the same polymer and amount of cross-linker. The only difference was that after blade coating the films, for the former, the solvent was let evaporate before cross-linking, while the latter was cross-linked immediately in solution. While the dielectric breakdown field of these two materials was the same, the latter gave a larger actuation. Material ***exo*****-E**_**62**_ showed a fast response and gave, after a few cycles, a reversible lateral actuation of 7% at about 15 V/μm even when the frequency was increased 10 times from 1 to 10 Hz (Figure 5b). Besides, this actuator did not indicate any sign of breakdown during and after more than 1100 actuation cycles. Thanks to the smaller elastic modulus of ***exo*****-E**_**62**_**′**, its lateral actuation strain was higher than that of ***exo*****-E**_**62**_ (12% at 15 V/μm vs 9.2% at 16 V μm^–1^, Figure S12b vs a). As another valid proof of reliability, material ***exo*****-E**_**62**_**′** survived more than 10000 actuation cycles, and only negligible hysteresis between the actuation cycles is observed (Figure 5c).

The actuators made from cross-linked bottlebrush polymers reported in the literature were tested using a small pressure. Therefore, the performance of the reported actuators is difficult to compare with the performance of our materials on which no pre-strain was applied. We were, however, curious to see how a small pressure applied to the actuator influences its performance. Therefore, another protocol (bulge test) was used for testing actuators constructed from ***mix*****-E**_**41**_, where a small pressure was applied (Figures S13 and S14). Actuation was calculated by analyzing the video of the actuator and simulating three-dimensional (3D) models resembling the film before and after actuation. At 1500 V, corresponding to about 18 V/μm, an areal actuation of 121% for ***mix*****-E**_**41**_ was measured. Note that the same material gave 10% lateral actuation when not pre-strained, representing 21% areal actuation at the same electric field.

**Scheme 2:**
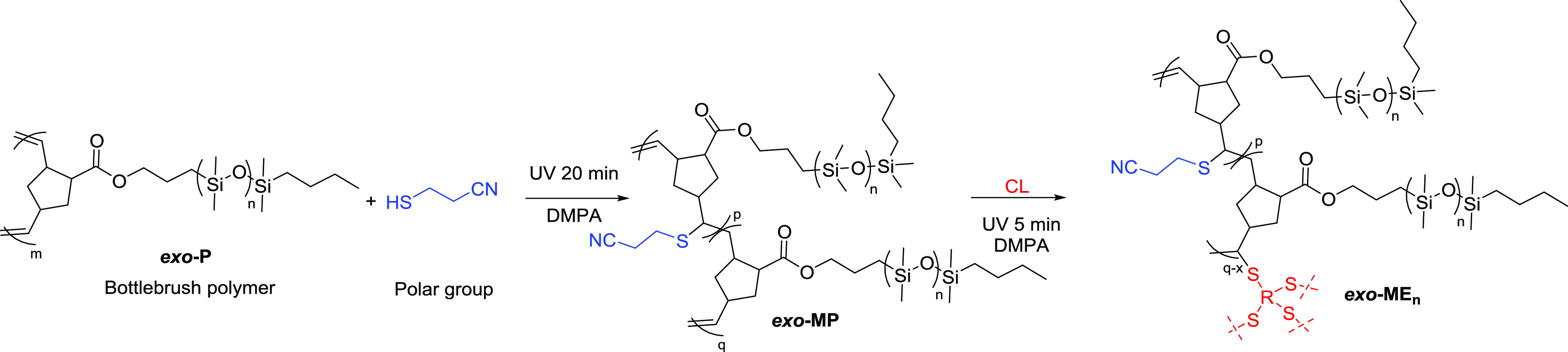
Synthetic Path to Polar Group Functionalized Bottlebrush Elastomers by the Thiol–Ene Reaction of 3-Mercapto Propionitrile with Double Bonds from ***exo*****-P** Backbone to Give ***exo*****-MP**

While the polynorbornene backbone seems to disfavor to some degree the strain-induced stiffening of our materials, its backbone has the great advantage of containing double bonds, which can be used in a post-polymerization modification. We therefore use a thiol–ene reaction to modify the bottlebrush polymer with polar 3-mercapto propionitrile (Scheme 2).

2,2-Dimethoxy-2-phenylacetophenone (DMPA) was used to initiate the reaction. The extent of the modification was determined by ^1^H NMR spectroscopy, which showed that approximately 16% of the double bonds reacted (Figure S15). The modified polymer ***exo*****-ME**_**n**_ was subsequently cross-linked using a similar protocol used for ***exo*****-E**_**n**_ to give materials ***exo*****-ME**_**n**_**′** (Table 4). The cross-linking was done immediately after casting to take advantage of the observed positive effect of solvent over the mechanical properties. For the nonpolar ***exo*****-E**_**n**_, the lowest molar concentration of −SH to achieve materials with good mechanical properties was 50 μmol/g; for the modified polymer ***exo*****-MP**_**n**_, the threshold decreased to 34 μmol/g, likely due to the dipolar interactions. The rather low amount of extractable confirms that the modified materials were efficiently cross-linked (Table 4). The obtained materials were evaluated in the tensile tests (Figure 2c) and the elastic modulus at different strain levels was calculated (Table 4). The elastic modulus of all materials increased significantly due to dipolar interactions of the nitrile groups.

**Table 4:** Mechanical Properties of ***exo*****-ME**_**n’**_ Silicone Elastomers Sample

entry	polymer used	–SH [μmol/g]^a^	–SH:double bonds^c^	*Y*_10%_ [kPa]	*Y*_50%_ [kPa]	*Y*_100%_ [kPa]	*s* [%]^d^	gel fraction [%]
***exo*****-ME**_**62**_**′**^b^	***exo*****-MP**	62	0.059	147 ± 3	144 ± 12	245 ± 45	88 ± 11	87
***exo*****-ME**_**50**_**′**^b^	***exo*****-MP**	50	0.048	127 ± 18	117 ± 12	154 ± 22	89 ± 18	87
***exo*****-ME**_**34**_**′**^b^	***exo*****-MP**	34	0.032	104 ± 13	88 ± 11	111 ± 13	104 ± 7	89

a Concentration of the thiol functional group to the mass of the polymer used.

b Polymer cross-linked with solvent.

c Molar ratio.

d Average strain at break of at least five samples.

Material ***exo*****-ME**_**62**_**′** showed the highest elastic modulus *Y*_10%_ = 147 kPa, which dropped to 144 kPa at 50% strain, but increased to 245 kPa at 100% strain. Similarly, the elastic modulus of material ***exo*****-ME**_**50**_**′** also dropped from 127 kPa at 10% strain to 117 kPa at 50% strain and grew to 154 kPa at 100% strain. In comparison, the elastic moduli of ***exo*****-E**_**62**_**′** and ***exo*****-E**_**50**_**′** at *Y*_10%_, *Y*_50%_, and *Y*_100%_ were smaller than their polar-group-modified counterparts. Furthermore, the small stiffening effect of ***exo*****-E**_**62**_**′** and ***exo*****-E**_**50**_**′** disappeared at 50% strain for the modified polymers. The softest material was ***exo*****-ME**_**34**_**′**, which had a *Y*_10%_ = 104 kPa, *Y*_50%_ = 88 kPa, and *Y*_100%_ = 111 kPa.

The dielectric properties of ***exo*****-ME**_**50**_**′** were also investigated and compared with ***exo*****-E**_**50**_**′** (Figure 3**c**). The increased permittivity to 5.5 reflects the chemical modification of ***exo*****-ME**_**50**_**′** with polar groups. This permittivity increase leaves the conductivity unaltered, while the dielectric loss decreases slightly.

Actuators constructed from material ***exo*****-ME**_**50**_**′** (32 μm thick) showed the largest lateral actuation of around 5.4% (11% area actuation) at 800 V (25 V/μm) (Figure S12f). The breakdown field of the actuators from this material was 27.7 V/μm. While the dielectric breakdown field of ***exo*****-ME**_**50**_**′** was increased, its largest actuation strain achieved was lower than ***exo*****-E**_**50**_**′**. Nevertheless, the maximum actuation pressure of the modified material ***exo*****-ME**_**50**_**′** increased significantly versus the unmodified materials ***exo*****-E**_**50**_**′** (375.62 vs 66.60 Pa). Although the polar-group-modified material did not show a larger actuation at lower voltages than ***exo*****-E**_**50**_**′**, the conducted experiments show the potential of this synthetic method, which can be used to synthesize bottlebrush polymers with increased dielectric permittivity.

## 3 Experimental Section

### 3.1 Materials

Polydimethylsiloxane, monohydride-terminated (**H-PDMS**, AB250915, viscosity 5–9 cSt., *M*_n_ = 150.8 Da, *M*_w_ = 387.3 Da, PDI = 2.57), was purchased from ABCR. Karstedt’s catalyst (platinum(0)-1,3-divinyl-1,1,3,3-tetramethyldisiloxane complex solution in xylene, Pt ≈ 2%), allyl alcohol, 4-(dimethylamino)pyridine (DMAP), *N*,*N*′-diisopropylcarbodiimide (DIC, 18.4 mL, 122.4 mmol, 3.4 eq.), 5-norbornene-2-carboxylic acid (mixture of *endo* and *exo*, predominantly *endo*), *exo*-5-norbornene carboxylic acid, first-generation Grubb’s catalyst, 2,2-dimethoxy-2-phenylacetophenone (DMPA), and pentaerythritol tetrakis(3-mercaptopropionate) (**CL**) were purchased from Sigma-Aldrich. Poly(vinyl alcohol) (PVA, R&G-PVA-Folientrennmittel) was purchased from Suter-Kunststoff AG. Methanol (MeOH), dichloromethane (DCM), ethyl acetate (EA), toluene (Tol), tetrahydrofuran (THF), and heptane were purchased from VWR. 3-Mercapto propionitrile was synthesized according to the literature.^49^ All chemicals were of reagent grade and used without purification; only toluene was dried over sodium using benzophenone as an indicator and DCM over calcium hydride and distilled prior to use.

### 3.2 Characterization

The gel fraction was determined by conducting extraction experiments with THF for 72 h. The THF solution was replaced by a fresh one, and the swollen materials were dried in a vacuum oven at 60 °C for 24 h until there was no change in mass. After repeating this procedure three times, the gel fraction of the material was calculated by dividing the mass of the material after the extraction experiment by the initial mass. More details about the characterization and equipment used can be found in the Supporting Information.

### 3.3 Synthesis of Mono-Hydroxyl-Terminated Poly(dimethylsiloxane), HO-PDMS

A mixture of polydimethylsiloxane, monohydride-terminated (**H-PDMS**, 60 g, 64 mmol), dry toluene (100 mL), allyl alcohol (A, 8.3 mL, 133 mmol), and Karstedt’s catalyst (1.6 mL) was reacted under argon at room temperature overnight. Then, the solvent was removed and the product was purified by column chromatography using a gradient of eluents of heptane:ethyl acetate = 50:1, 10:1, and 2:1, *R*_f_ value of the product was about 0.6 in heptane:ethyl acetate = 10:1.

### 3.4 Synthesis of Macromonomers

The reaction flask was dried by a heating gun before use. A mixture of 4-(dimethylamino)pyridine (DMAP, 14.6 g, 122.4 mmol, 3.4 equiv), norbornene acid (11.67 g, 82.8 mmol, 2.3 equiv), *N*,*N*′-diisopropylcarbodiimide (DIC, 18.4 mL, 122.4 mmol, 3.4 equiv), and dry DCM (120 mL) was stirred for 2 h at RT. The alcohol-terminated PDMS (**HO-PDMS**, 34 g, 36 mmol, 1 equiv) was added, and the mixture was stirred at rt for 24 h. The reaction was monitored by thin-layer chromatography. After all starting polymer was consumed, the reaction mixture was purified by column chromatography. The *R*_f_ value of the product spot was 0.6–0.5 using the mixture of heptane:ethyl acetate = 10: 1. The macromonomer ***mix*****-M** was prepared following the general procedure by using 5-norbornene-2-carboxylic acid, a mixture of *endo* and *exo*, predominantly *endo* (C1). The macromonomer ***exo*****-M** was prepared following the general procedure by using *exo*-5-norbornene carboxylic acid (C2).

### 3.5 Synthesis of Bottlebrush Polymer ***mix*****-P** and ***exo*****-P**

The macromonomer, ***mix*****-M** or ***exo*****-M** (20 g, ∼21.26 mmol, 800 eq.), was put in a flask and backfilled with Ar, followed by the addition of dry DCM (250 mL). Then, the mixture was degassed three times, followed by the addition of the first-generation Grubb’s catalyst (23 mg, 26.6 μmol, 1 equiv). The mixture reacted under different temperatures and duration depending on the macromonomer type. Monitored by TLC, after the full consumption of the starting polymer, ethyl vinyl ether (2 mL) was added to quench the reaction. Next, the product was purified by precipitation from DCM to MeOH. After the purification, the bottlebrush polymer (***mix*****-P** or ***exo*****-P**) was kept as a solution of toluene with a concentration between 300–600 mg/mL. The bottlebrush polymer ***mix*****-P** was prepared following the general procedure using macromonomer ***mix*****-M** and heating to reflux at 65 °C for 12 h. The bottlebrush polymer ***exo*****-P** was prepared following the general procedure by using macromonomer ***exo*****-M** and heating at 40 °C for 2 h. The bottlebrush polymer ***exo*****-P**′ was prepared following the general procedure by using macromonomer ***exo*****-M** and heating at 40 °C for 3.5 h.

### 3.6 Preparation of Material ***mix*****-E_n_** and ***exo*****-E_n_**

A solution of **CL** (10%, v/v) in toluene was prepared. A mixture of the corresponding amount of bottlebrush polymer solution, **CL** (for the amount used, see Tables 2), and 2,2-dimethoxy-2-phenylacetophenone (DMPA, 2 wt % to bottlebrush polymer) was put in a vial and mixed well by centrifugation. Next, the mixture was put on a Teflon substrate and cast by a doctor blade with a certain thickness, whereas the films for electromechanical tests were cross-linked on PVA substrates (see the Supporting Information). The PVA film, on which the dielectric films were cross-linked, can be easily peeled off from the glass substrate. Thus, no defects are produced in the dielectric films during manipulation, as the films to be tested are not touched by hand. After blade coating the solution mixture, the solvent was let evaporate for 8 h and then irradiated for 5-min UV to give cross-linked films. Only the films of materials ***exo*****-E**_**62**_′ and ***exo*****-E**_**50**_′ were cross-linked immediately after casting from the solution. Before the characterization, all of the materials were put in a vacuum oven at 60 °C overnight to remove the residual solvents.

### 3.7 Synthesis of ***exo*****-MP**

The mixture of macromonomer, ***exo*****-M** (10 g, ∼10.0 mmol, 1 equiv), 3-mercapto propionitrile (1.776 g, 20 mmol, 2 equiv), and DMPA (20 mg, 0.1 mmol, 1 mol %) was put in a flask and degassed and backfilled with Ar for three times, followed by UV irradiation for 20 min. Next, the product was purified by precipitation from DCM with MeOH. After the purification, the bottlebrush polymer (***exo*****-MP**) was kept as a toluene solution with a concentration between 300 and 600 mg/mL.

### 3.8 Synthesis of ***exo*****-ME**_**n**_

The synthesis of material ***exo*****-ME**_**n**_ is the same as that of ***mix*****-E**_**n**_ and ***exo*****-E**_**n**_. A solution of **CL** (10%, v/v) in toluene was prepared. A mixture of the corresponding amount of bottlebrush polymer solution, **CL** (for the amount used, see Tables 4), and 2,2-dimethoxy-2-phenylacetophenone (DMPA, 2 wt % to bottlebrush polymer) was put in a vial and mixed well by centrifugation. Next, the mixture was put on a Teflon substrate and cast by a doctor blade with a certain thickness, whereas the films for electromechanical tests were cross-linked on PVA substrates. After blade coating the solution mixture, it was irradiated for 5 min UV to give cross-linked films. Before the characterization, all of the materials were put in a vacuum oven at 60 °C overnight to remove the residual solvents.

## 4 Conclusions

We reported a series of soft dielectric elastomers based on cross-linked bottlebrush polymers prepared by ROMP of norbornene macromonomers followed by cross-linking *via* thiol–ene reaction. The highly efficient and fast thiol–ene reaction allows the formation of defect-free thin films in the air. Materials prepared using the same amount and type of reagents showed different properties when cross-linked in the solution and after solvent removal. Those cross-linked in solution were softer and showed a small strain stiffening effect. All materials show ultralow elastic modulus and mechanical loss factors below 0.1, low dielectric loss factor below 0.05, and very low conductivity of 5 × 10^–14^ S/cm. The best material gave 12% lateral actuation strain at 1000 V when no pre-strain was applied. Additionally, actuators constructed with these soft materials survived up to 10,000 cycles at different frequencies in the cyclic electromechanical test. Furthermore, the double bonds of the polymer backbone can be used for cross-linking and also allow chemical modification with polar groups, thus achieving materials with increased dielectric permittivity. This could lead to actuators with lower driving voltage and increased actuation pressure.

## Supporting Information Available

^1^H NMR spectra of monohydride- and mono-hydroxyl-terminated poly(dimethylsiloxane) (top) and ^13^C NMR spectrum of alcohol-terminated PDMS (bottom) in CDCl_3_ (Figure S1); ^1^H NMR spectra of starting mix-norbornene carboxylic acid, macromonomer ***mix*-M** and of the starting alcohol-terminated PDMS and ^13^C NMR spectrum of macromonomer ***mix*****-M** in CDCl_3_ in CDCl_3_ (Figure S2); and ^1^H and ^13^C NMR spectra of macromonomer ***exo*-M** in CDCl_3_ (Figure S3) (PDF)
