# Supplementary material for: On-Demand Cross-Linkable Bottlebrush Polymers for Voltage-Driven Artificial Muscles
Source: ACS Appl Mater Interfaces. 2023 Apr 12;15(16):20410–20. doi: 10.1021/acsami.2c23026 (PMC10141291; doi:10.1021/acsami.2c23026)
Supplement: Supplementary file 1 — am2c23026_si_001.pdf [file am2c23026_si_001.pdf]

Supporting information

On-demand cross-linkable bottlebrush polymers for voltage-driven artificial muscles

*Yeerlan Adeli,[a,b] Francis Owusu, [a,b] Frank A. Nüesch,[a,b] Dorina M. Opris[a]**

[a] Laboratory for Functional Polymers, Swiss Federal Laboratories for Materials Science and Technology Empa, Ueberlandstr. 129, CH-8600, Dübendorf, Switzerland

[b]Institute of Chemical Sciences and Engineering, Ecole Polytechnique Federale de Lausanne, EPFL, Station 6, CH-1015 Lausanne, Switzerland

E-mail: dorina.opris@empa.ch

EXPERIMENTAL SECTION

^{1}H and C^{13} NMR spectra were recorded with a Bruker AVANCE 400 NMR spectrometer using a 5 mm BBO ProdigyTM CryoProbe at 400.18 and 100.63 MHz, respectively. Chemical shifts (δ) in ppm are calibrated to the residual solvent peak ($CDCl_3$: δ = 7.26; 77.16).

Gel permeation chromatograms were recorded using an Agilent 1100 Series HPLC (columns: serial coupled PSS SDV 5 m, 100 Å and PSS SDV 5 m, 1000 Å, detector: DAD, 235 and 360 nm; refractive index), with THF as the mobile phase. PDMS standards were used for calibration and toluene as an internal standard.

Thermogravimetric analysis (TGA) of ***mix*-$\mathbf{P_n}$** and ***exo*-$\mathbf{P_n}$** was conducted with a PerkinElmer TGA7 in either a nitrogen or helium atmosphere by heating the sample from 30 to 900 °C at a speed of 20 °C/min.

Differential scanning calorimetry (DSC) investigations were under taken on a Pyris Diamond DSC (PerkinElmer USA) instrument under nitrogen flow (50 mL × min^{-1}), in aluminum crucibles with pierced lids and using about 10 mg sample mass.

The tensile tests were performed using a Zwick Z010 tensile test machine with a cross-head speed of 50 mm min^{-1}. Tensile test specimens with a gauge width of 2 mm and a gauge length of 18 mm were prepared by die-cutting. The strain was determined using a longitudinal strain extensometer. The curves were averaged from different independent experiments. The Young's modulus at 10% strain was determined from the slope of the stress−strain curve using a linear fit to the data points from 0 to 10% strain, while for the Young's moduli at 50 and 100%, a linear fit to the data points from 40 to 50% and from 90 to 100% strain, respectively, was used.

Dynamic mechanical analysis was carried out on a RSA 3 DMA from TA Instruments. Stripes of 10 mm × 20 mm were measured under a dynamic load of 2 g, at 2% strain in the frequency range of 0.1−10 Hz at 25 °C. The mechanical loss factor tan (δ) is given as the fraction of imaginary and real storage modulus at 2% strain.

Dielectric measurements were performed in the frequency range from 1 to 10^6 Hz using a Novocontrol Alpha-A frequency analyzer. The VRMS (root mean square voltage) of the probing ac electric signal applied to the samples was 1 V. The permittivity ε' was determined from the capacitance $C = \varepsilon'\varepsilon_0 A/d$, where *A* is the electrode area, *d* is the thickness of the film, and ε_0 is the vacuum permittivity. The thickness of the film was measured by a micrometer gauge with an uncertainty of ±5 µm. The samples were prepared by placing the film between two stainless steel discs with a diameter of 20 mm. Before measurement, the samples were annealed at 80 °C in a vacuum oven.

Electromechanical tests were performed using circular membrane actuators at ambient temperature and humidity. Before crosslinking the bottlebrush polymer, a substrate was made by putting and casting PVA with a thickness of 400 μm on a glass substrate. After PVA solidified in 1 hour, the mixture of bottlebrush polymer was cast on the PVA substrate and cross-linked. Then the films together with the PVA substrate were fixed between two circular rigid frames with an inner diameter of 25 mm. To remove the PVA substrate, the fixed film and circular rigid frames were put into de-ionized warm water with 60 °C. The water was constantly changed every hour. After about 6 h, the fixed film and circular rigid frames were put into vacuum oven with 60 °C to remove residual solvent and water. Circular electrodes (8 mm diameter) of carbon black powder was applied to each side of the film. A FUG HCL- 35-12,500 high voltage source served as a power supply for actuator tests. We gradually increase the voltage by 100 V and up to 1700 V. The actuation strain was measured optically as the extension of the diameter of the electrode area via a digital camera, using an edge detection tool of a LabView program to detect the boundary between the black electrode area and the transparent film.

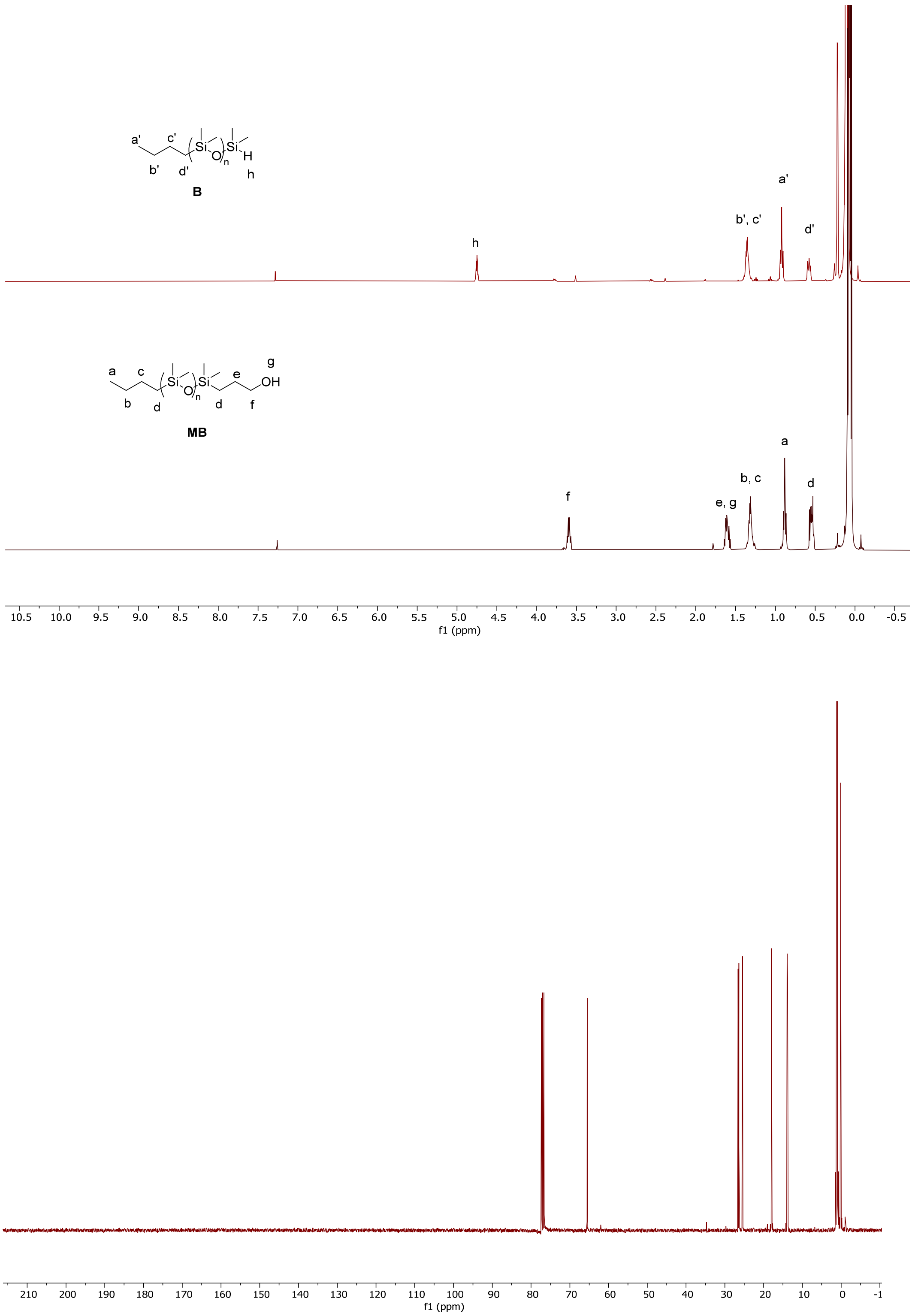

Figure S1. ^{1}H NMR spectra of mono-hydride and mono-hydroxyl terminated polydimethylsiloxane (top) and ^{13}C NMR spectrum of alcohol terminated PDMS (bottom) in $CDCl_3$.

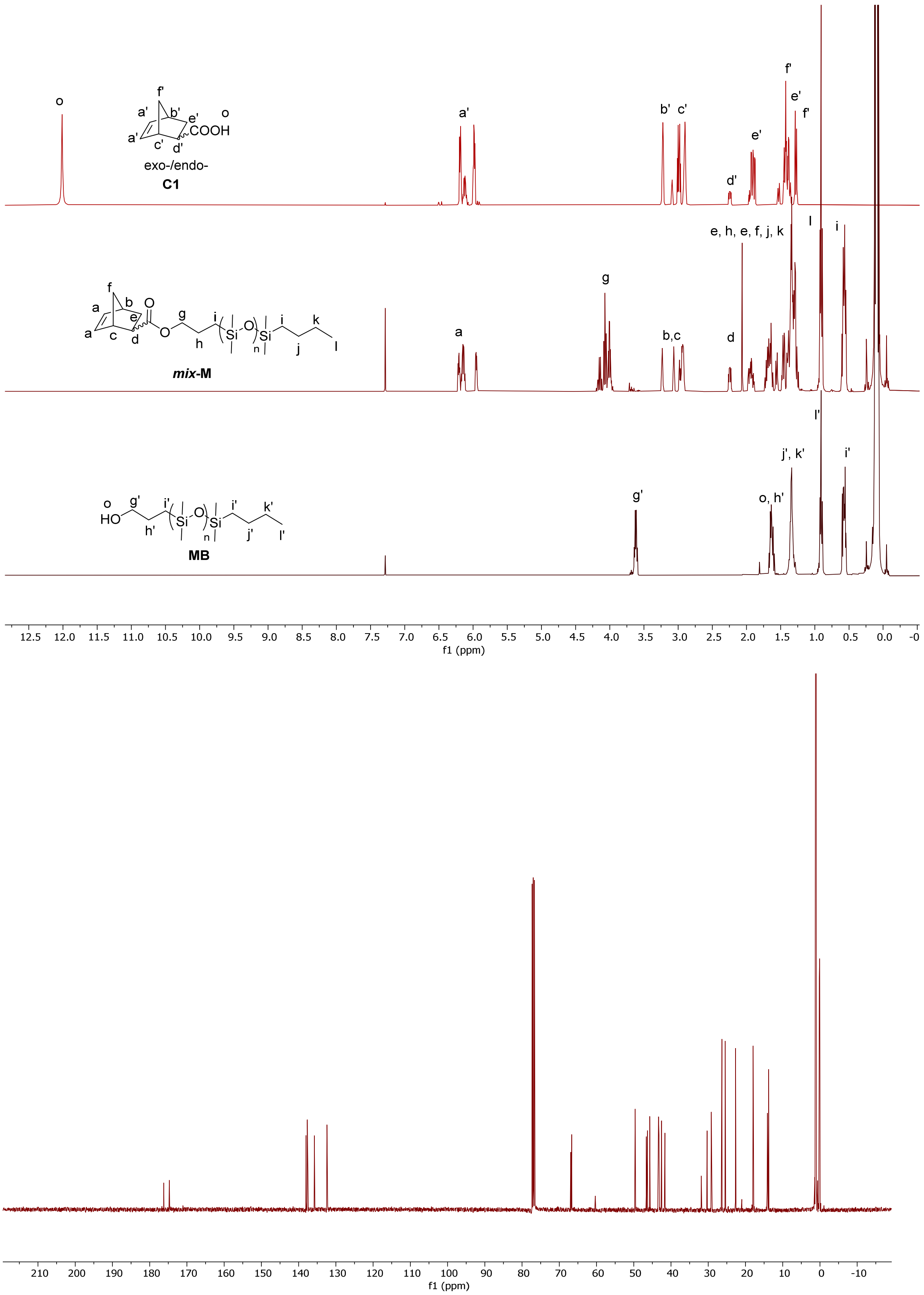

Figure S2. ^{1}H NMR spectra of starting mix-norbornene carboxylic acid, macromonomer ***mix*-M** and of the starting alcohol terminated PDMS (top) and ^{13}C NMR spectrum of macromonomer ***mix*-M** in $CDCl_3$ (bottom) in $CDCl_3$.

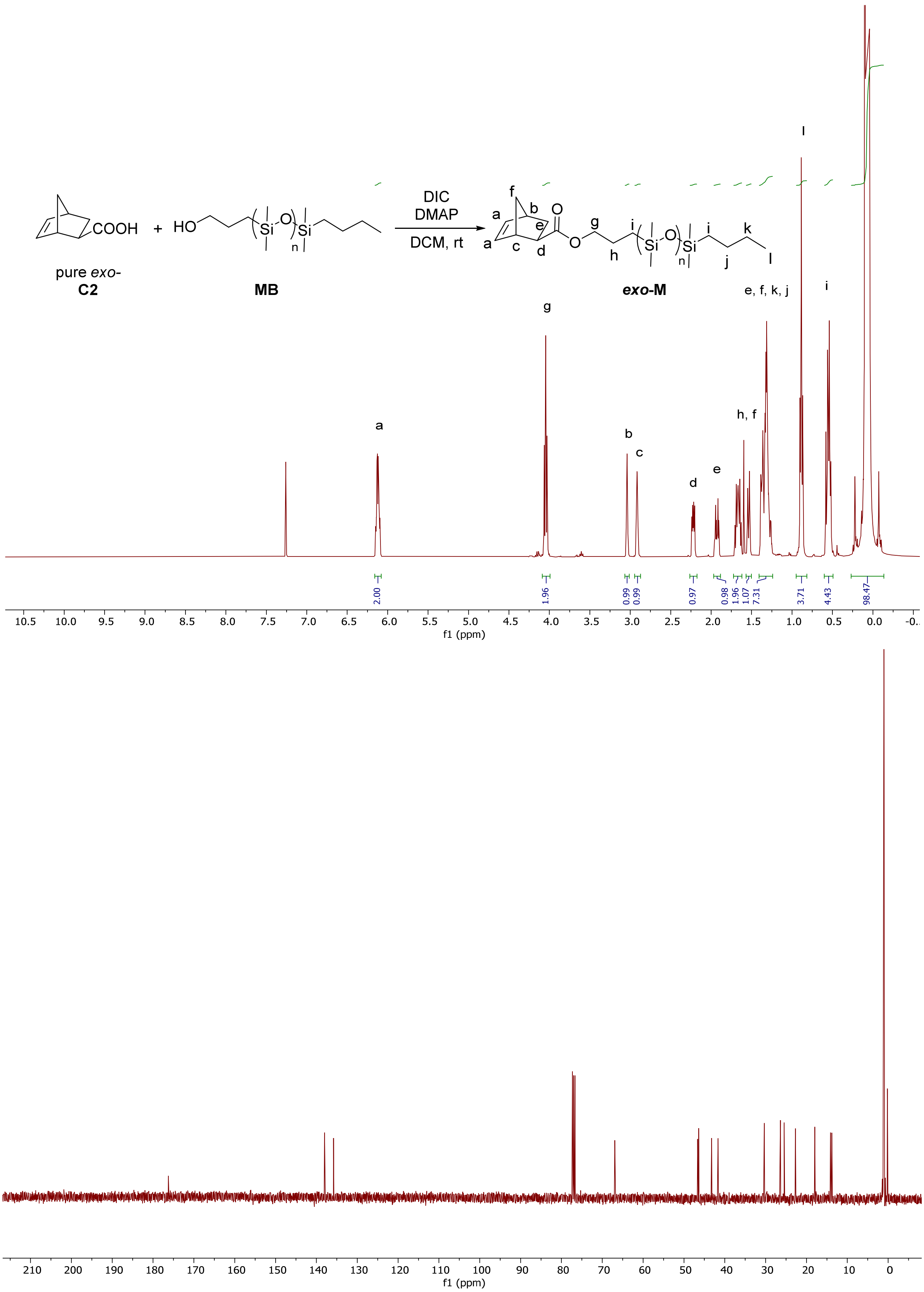

Figure S3. ^{1}H (top) and ^{13}C (bottom) NMR spectra of macromonomer ***exo*-M** in $CDCl_3$.

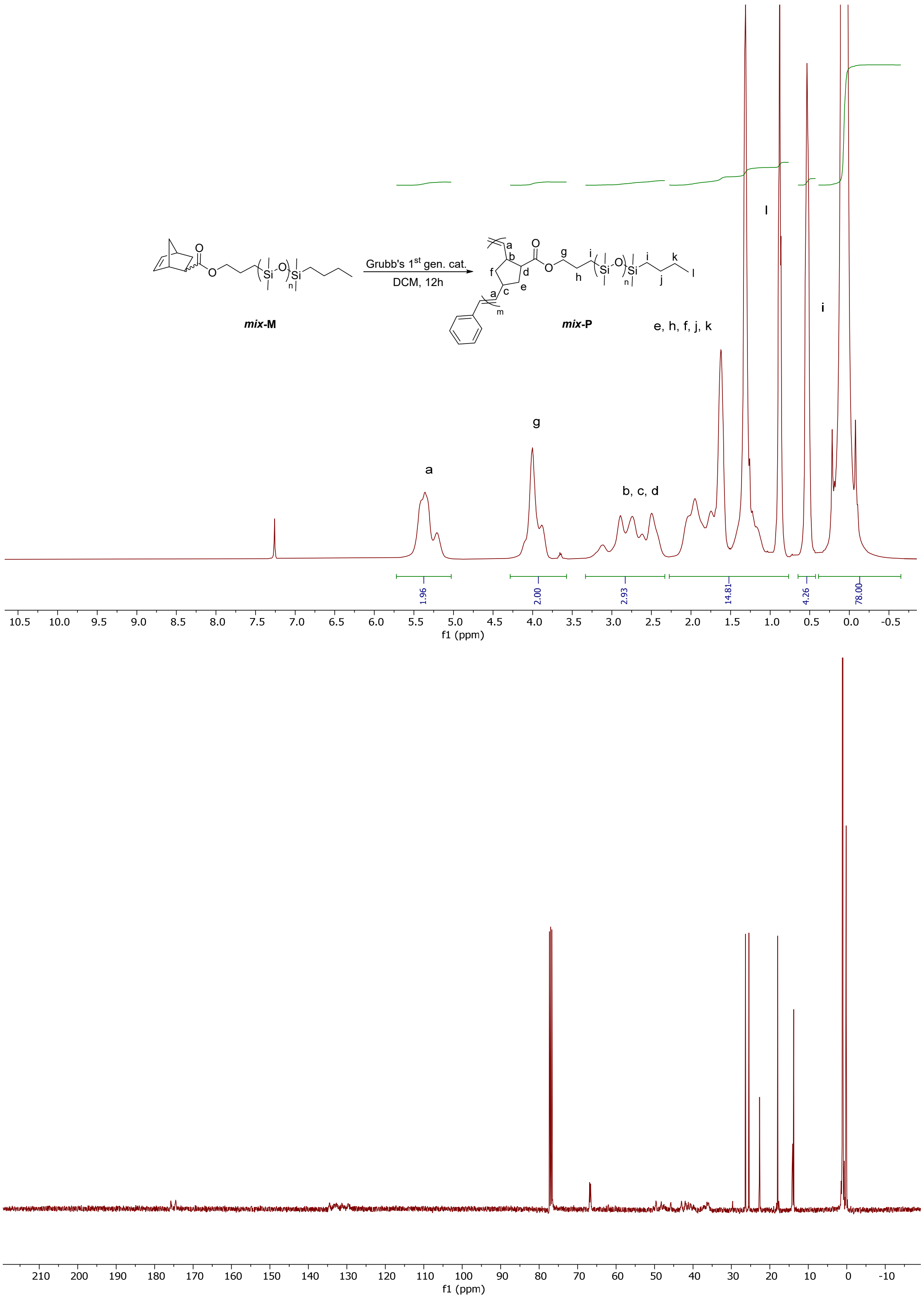

Figure S4. ^{1}H (top) and ^{13}C (bottom) NMR spectra of bottlebrush polymer ***mix*-P** in $CDCl_3$.

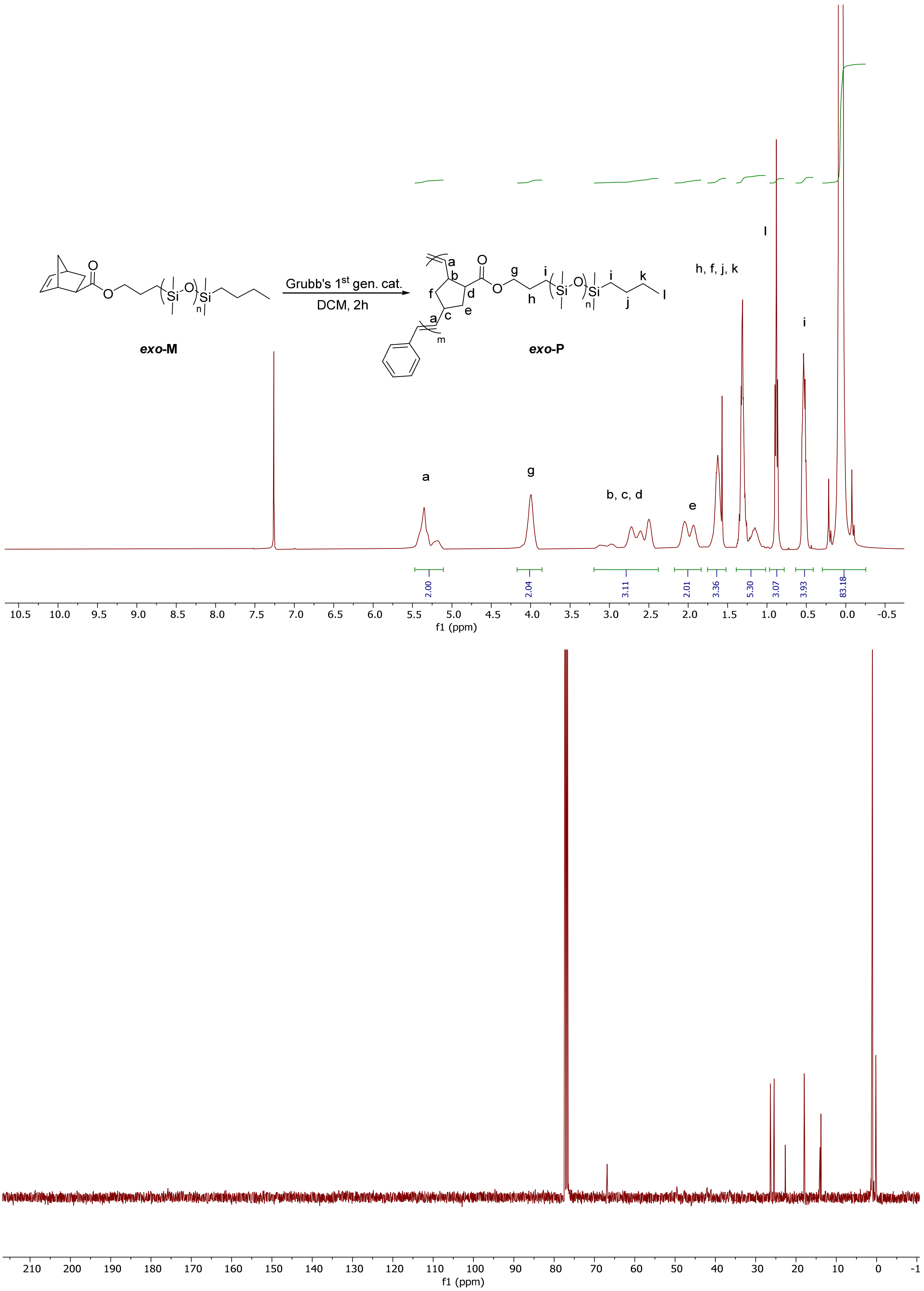

Figure S5. ^{1}H (top) and ^{13}C (bottom) NMR spectra of bottlebrush polymer ***exo*-P** in $CDCl_3$.

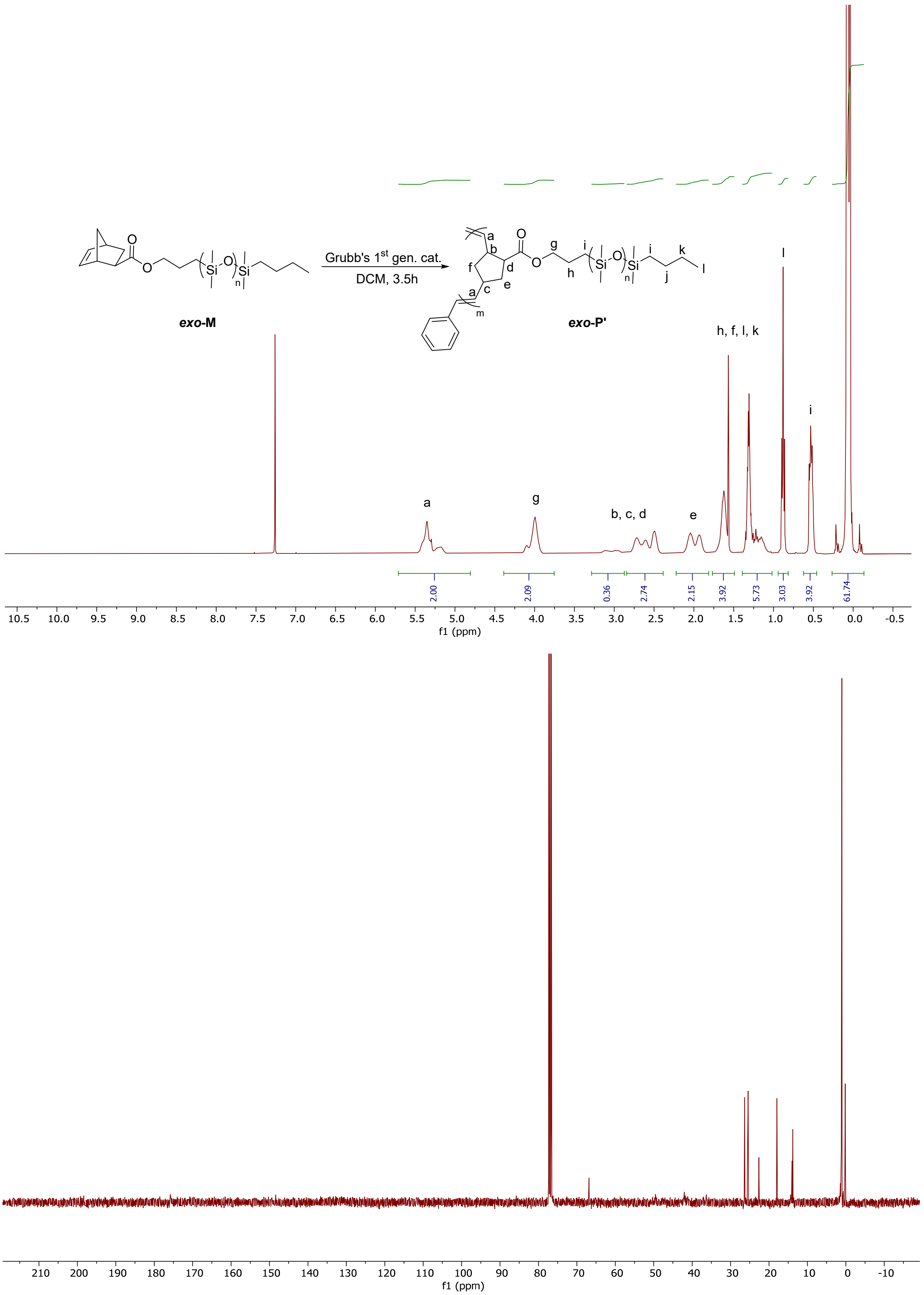

Figure S6. ^{1}H (top) and ^{13}C (bottom) NMR spectra of bottlebrush polymer ***exo*-P'** in $CDCl_3$.

Table S1. The molar masses and molar masses distributions results from GPC characterization.

Entry	M_n [Da]	M_w [Da]	PDI
**H-PDMS**	192	477	2.48
**HO-PDMS**	201	466	2.31
***mix*-M**	182	346	1.90
***exo*-M**	187	526	2.81
***mix*-P**	23650	38060	1.61
***exo*-P**	33170	74380	2.24
***exo*-P'**	271200	750030	2.76
***exo*-MP**	137630	193200	1.40

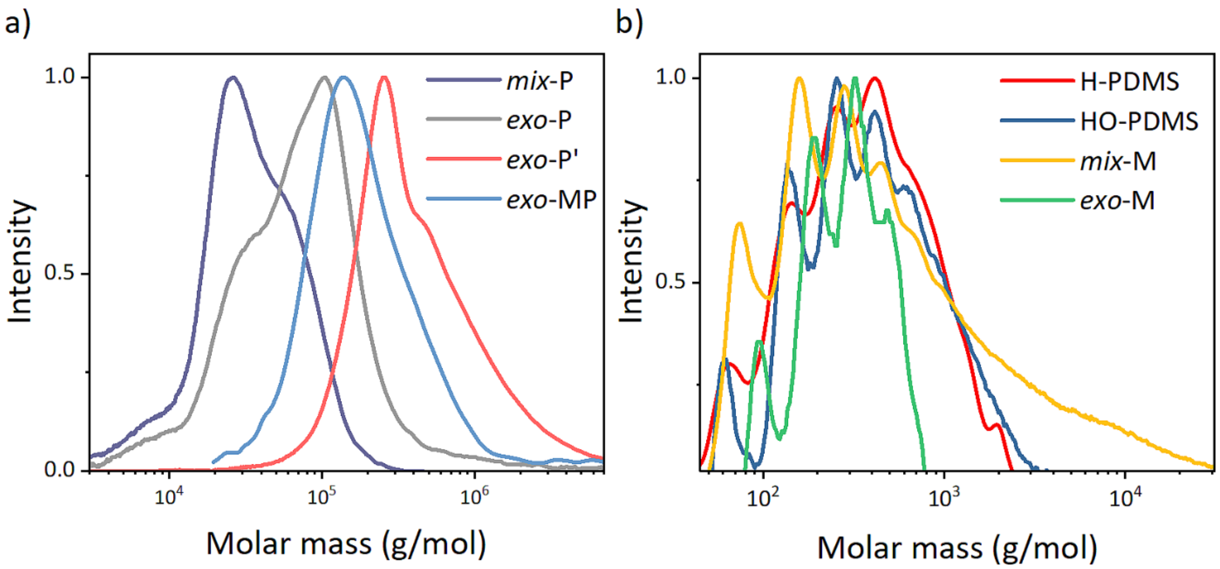

Figure S7. GPC eluogram of ***mix*-P**, ***exo*-P**, ***exo*-P'** (a) and of **exo-MP H-PDMS**, **HO-PDMS**, ***mix*-M** and ***exo*-M** (b).

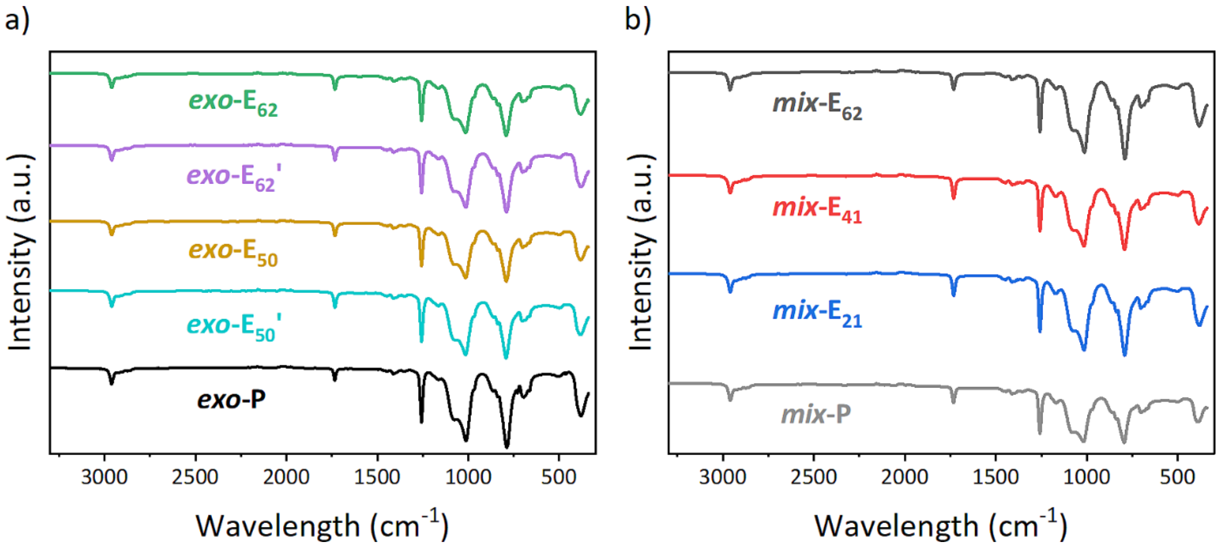

Figure S8. IR spectra of the materials made from bottlebrush polymer ***mix*-P** (a), and ***exo*-P** (b).

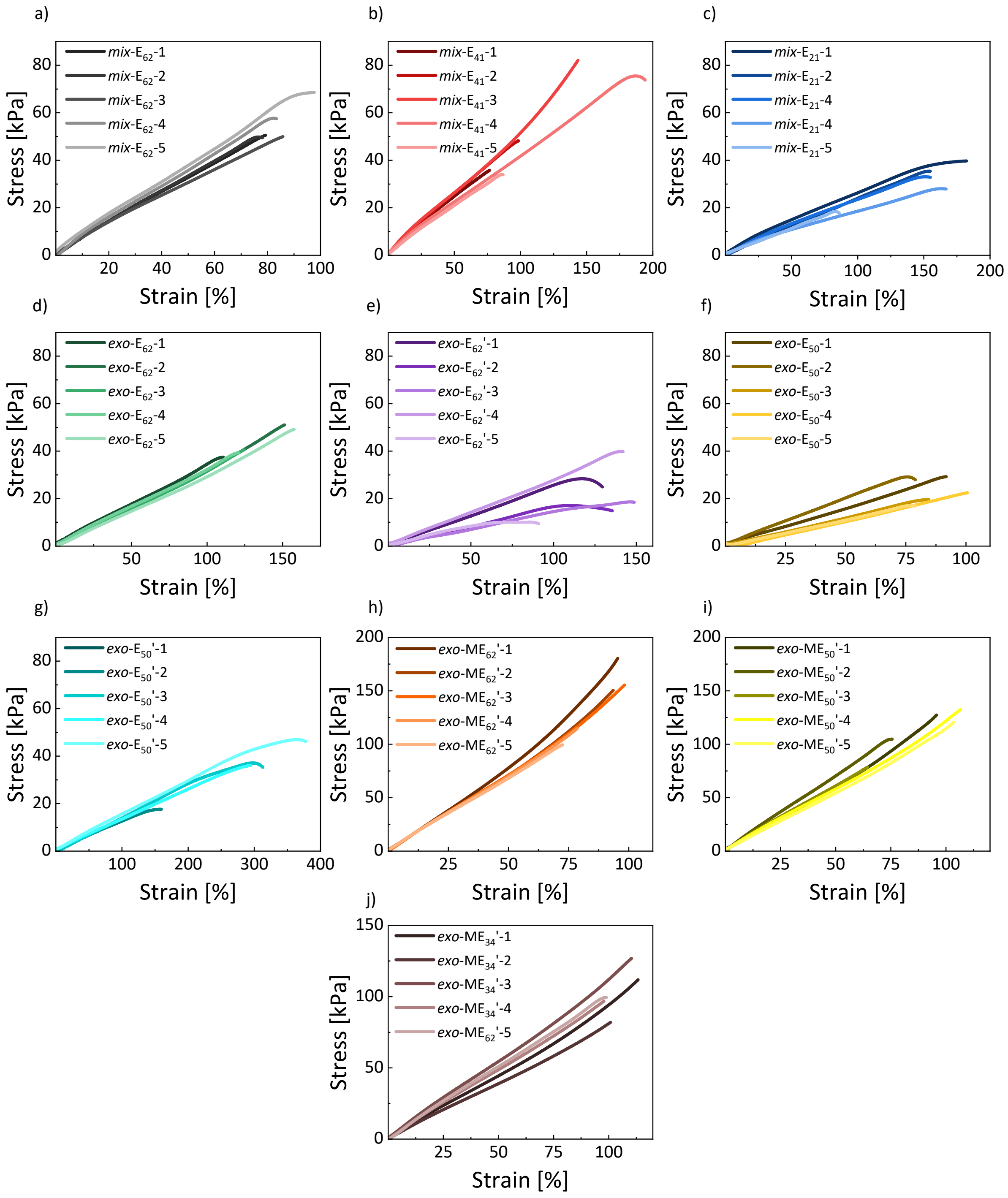

Figure S9. Stress-strain curves of materials ***mix*-E$_n$** (a to c), ***exo*-E$_n$** (d to g), and ***exo*-ME$_n$'** (h-i).

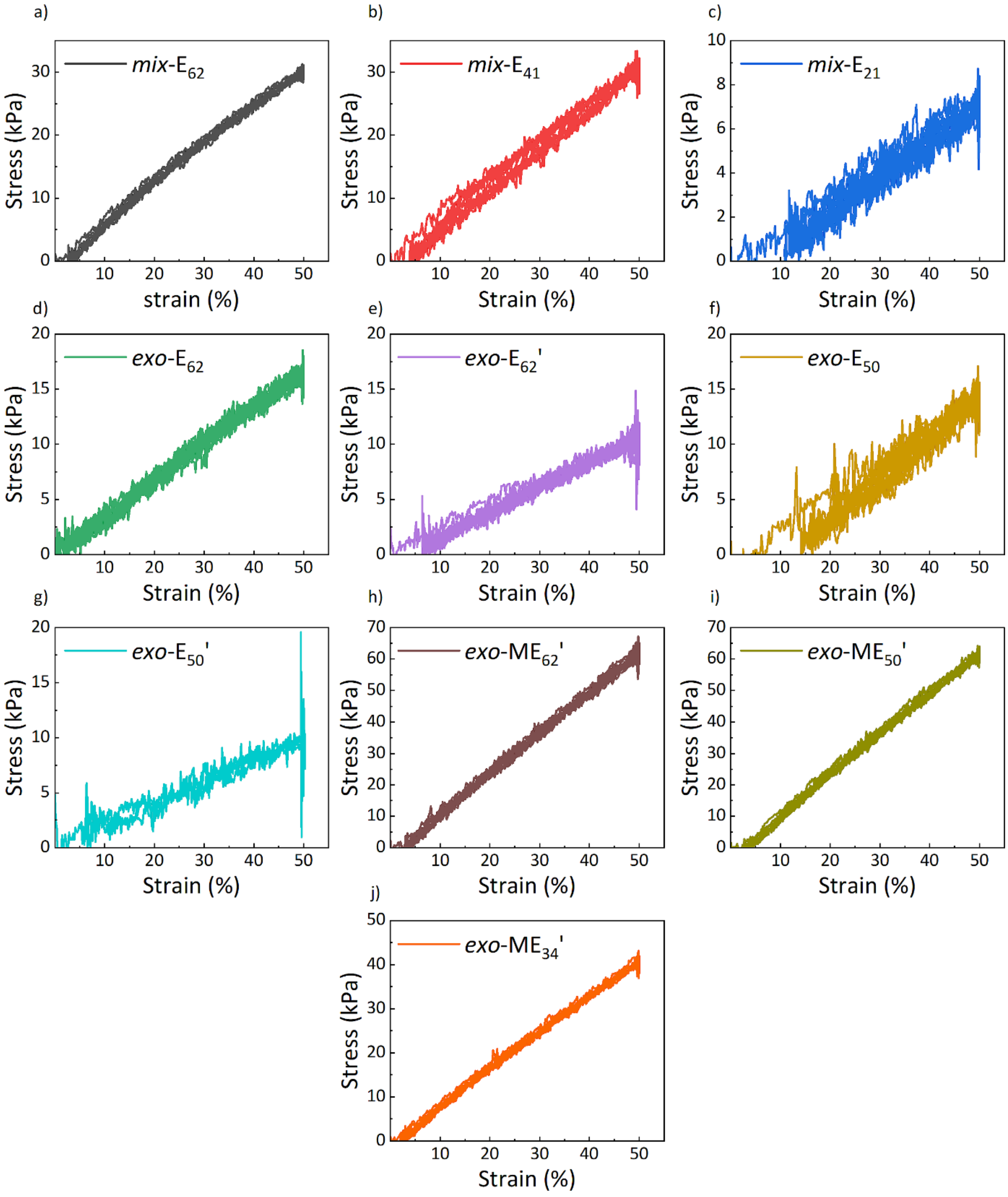

Figure S10. Stress−strain relaxation curves up to 50% strain of materials ***mix*-E$_n$** (a to c), ***exo*-E$_n$** (d to g), and ***exo*-ME$_n$'** (h-i)..

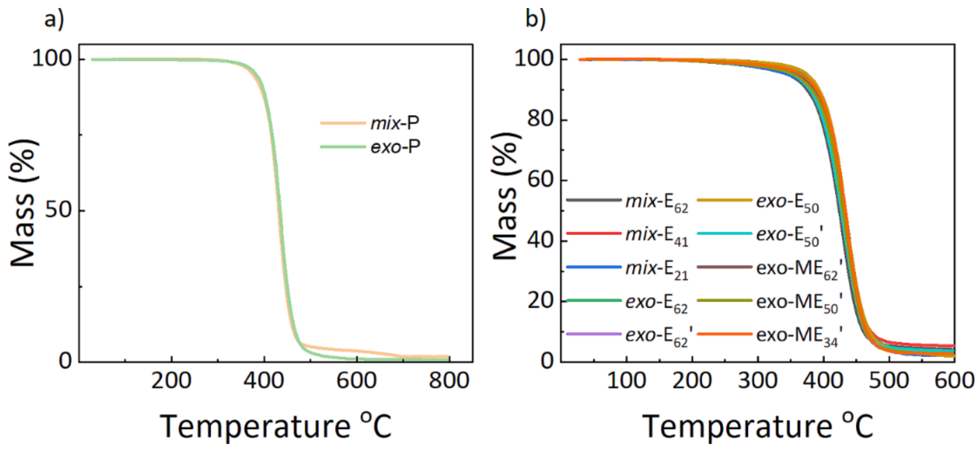

Figure S11. TGA graph of bottlebrush polymer ***mix*-P** and ***exo*-P** (a), and the materials made from bottlebrush polymer ***mix*-P**, ***exo*-P** and ***exo*-MP** (b).

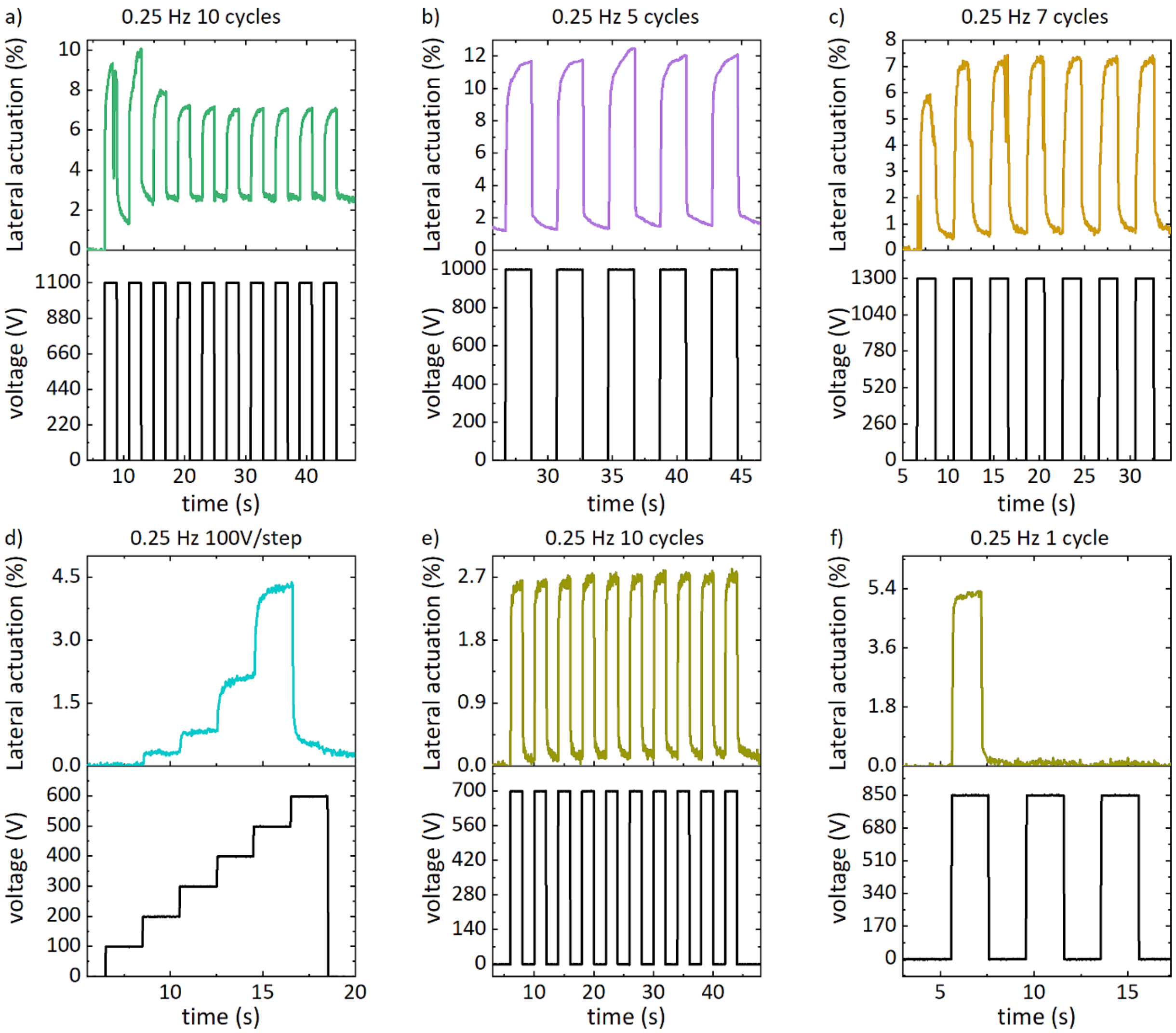

Figure S12. DEA measurements of the free-standing thin films for materials ***exo*-E$_{62}$** (67 µm thickness, a), ***exo*-E$_{62}$'** (66 µm thickness,b), ***exo*-E$_{50}$** (61 µm thickness, c), ***exo*-E$_{50}$'** (51 µm thickness, d), ***exo*-ME$_{50}$'** (35 µm thickness, e) and ***exo*-ME$_{50}$'** (46 µm thickness, f). Actuator with a small applied pressure (airbag actuator)

Procedures about the airbag actuator. The airbag actuator was assembled according to **Figure S13**. A compressible elastic ring with outer and inner diameters of 40 and 10 mm, respectively, was made from commercial VHB foil and placed on top of the DEA (made from ***mix*-E$_{42}$**) chamber, allowing the active actuator part to actuate outside the chamber. The DEA chamber consists of an ensemble of layers: circular VHB film with the protective film on, a VHB ring (outer and inner diameters of 40 and 35 mm, respectively), a circular rigid frame ((outer and inner diameters of 40 and 35 mm, respectively), VHB ring ((outer and inner diameters of 40 and 35 mm, respectively), and the DEA membrane. The electrical contact with the actuator was done with Al stripes. Finally, a PMMA round plate with a diameter of 45 mm was applied from the bottom and a plastic ring with inner and outer diameters of 25 and 45 mm was applied from the top, and then the entire ensemble was fixed with two clamps. The clamps created a uniform pressure on the actuator. During the measurement, the power supplier measured the voltage and a camera recorded a video of the actuator. Afterward, the actuation analysis

was processed by software Photoshop to measure the parameters according to the scale bar taken from the thickness of the white rigid frame. Then by software CorelDraw, the determination of the dome's geometry was accomplished using the side-profile image, which was captured by Photoshop. Thereafter, by simulation of software Autodesk fusion 360, the actuation surfaces before and after actuation were constructed and surface areas were calculated.

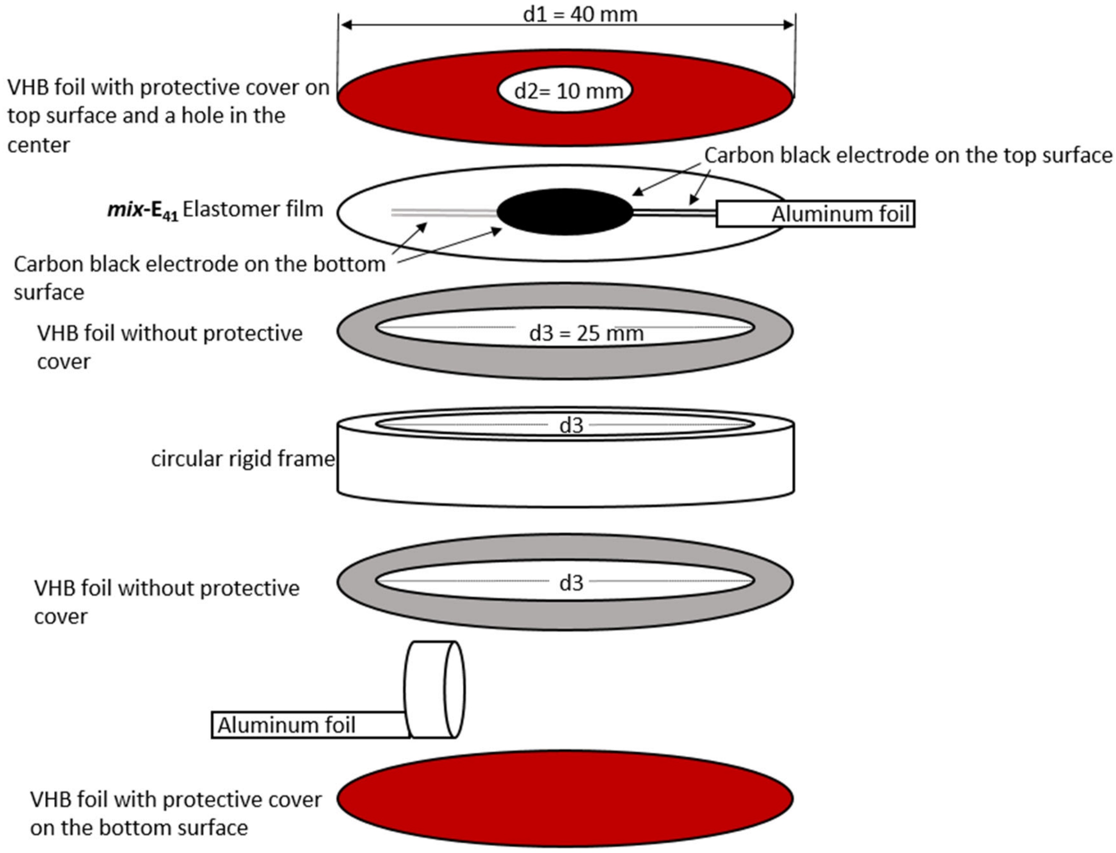

Figure S13. The structure of airbag actuator ***mix*-E$_{41}$**.

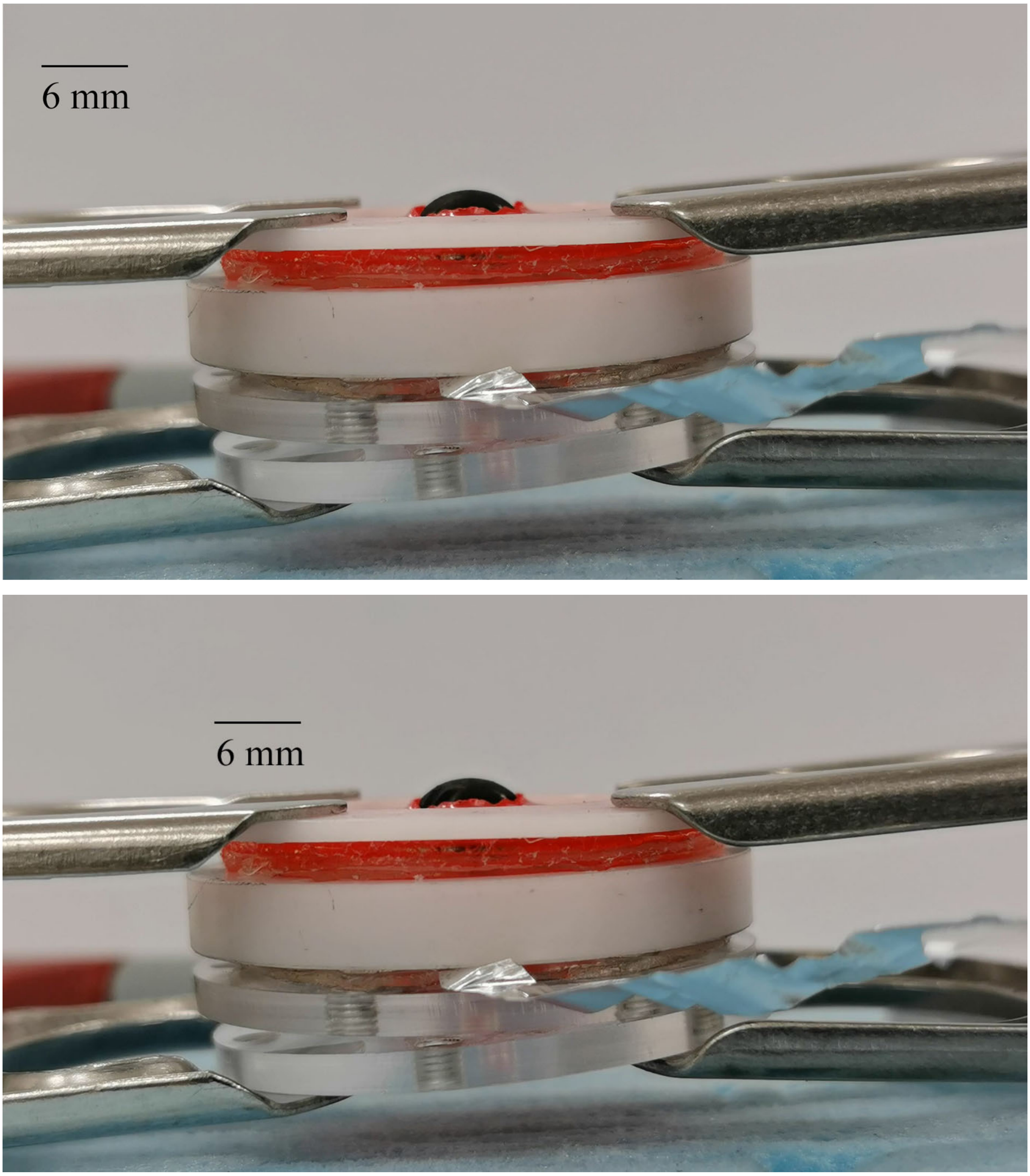

Figure S14. Airbag actuator made from a thin film from ***mix*-E$_{62}$**, voltage off state (top), and voltage on the state (1500V, bottom).

The original film thickness was 84 µm, therefore, considering the corresponding thickness, the breakdown field of this airbag actuator was 21.6 V/ µm.

The extent of polar group modification was determined by the ratio between integration from the vinylene group protons' signal (a) and –C(O)O-CH_2- group's signal (g), which equaled to 16% (Figure S15).

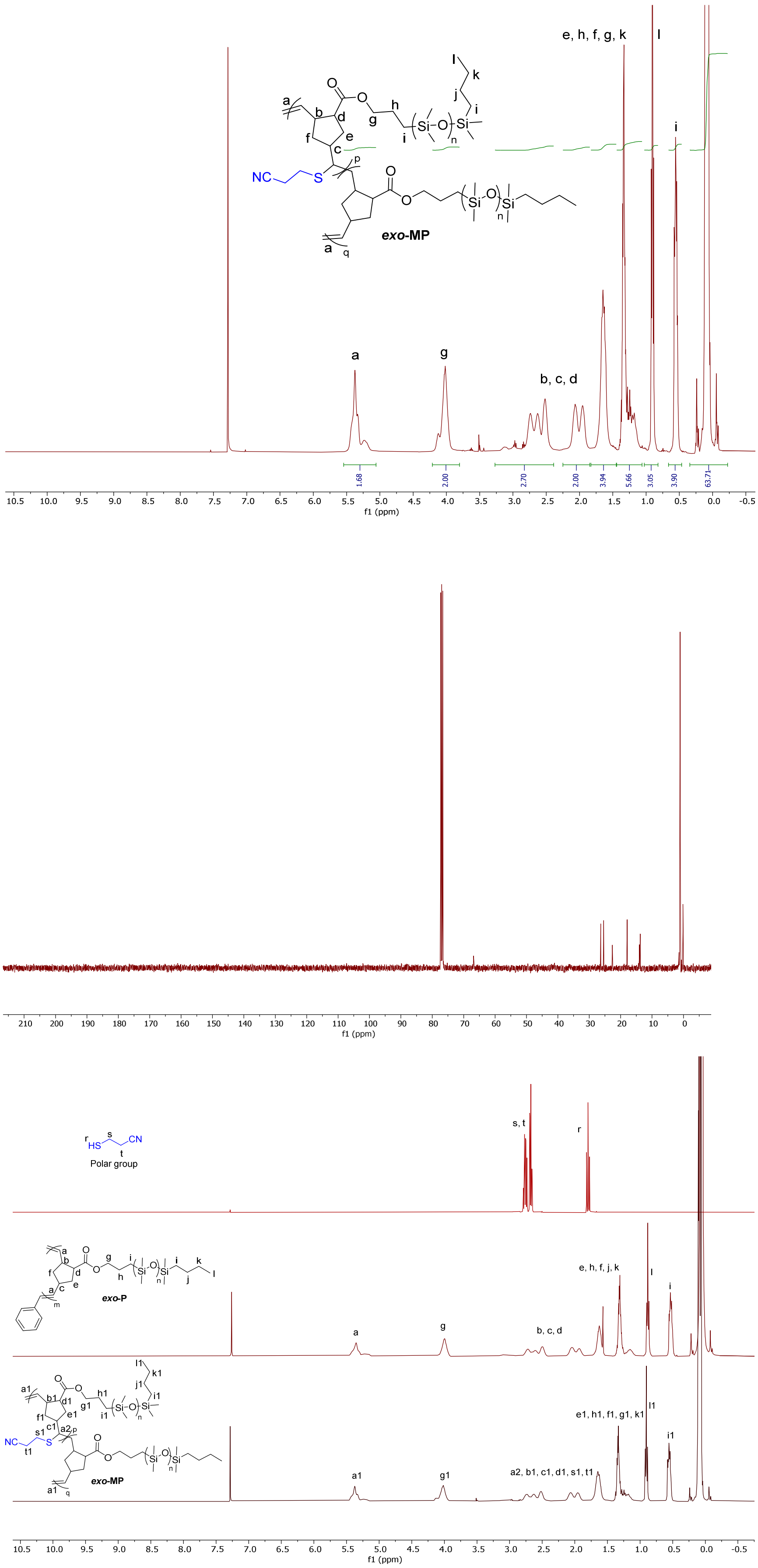

Figure S15. ^{1}H (top) and ^{13}C (middle) NMR spectra of polar group modified bottlebrush polymer ***exo*-MP** in $CDCl_3$, and stacked ^{1}H NMR spectra of polar group, ***exo*-P**, and ***exo*-MP** in $CDCl_3$ (bottom).
